# Prediction of Standard Combustion Enthalpy of Organic Compounds Combining Machine Learning and Chemical Graph Theory: A Strategy

**DOI:** 10.1021/acsomega.5c05927

**Published:** 2025-09-08

**Authors:** Fernanda Saviñon-Flores, Jesús A. Arzola-Flores, Miguel A. García-Castro, Fausto Díaz-Sánchez, Esmeralda Vidal Robles, Fidel Aaron Maruri Valderrabano

**Affiliations:** Facultad de Ingeniería Química de la Benemérita Universidad Autónoma de Puebla, 18 Sur y Avenue San Claudio, C.P., Puebla, Pue 72570, México

## Abstract

The prediction of thermochemical properties such as the standard enthalpy of combustion is essential for the design and evaluation of energetic materials. In this study, the prediction of this thermochemical property is proposed through a QSPR strategy that combines machine learning and chemical graph theory. The data set consisted of 3477 organic compounds. SMILES codes were used for each molecule to construct their molecular graphs, from which topological indices such as Estrada, Wiener, and Gutman, as well as centrality measures, were calculated. These descriptors served as predictors in supervised learning models, with tree-based ensemble models showing the best performance. The best-performing model, random forest, achieved the following metrics on the test set: *R* ^2^ = 0.9810, *MAE* = 287.5988 kJ·mol^–1^, *MAPE* = 0.1048, *RMSE* = 551.9050 kJ·mol^–1^, and *RMSLE* = 0.1933. Interpretability analysis using SHAP confirmed that the Estrada and Gutman indices were the most influential variables in the predictions. In addition, the same random forest model was trained using 210 molecular descriptors obtained from RDKit, yielding slightly better metrics: *R* ^2^ = 0.9927, *MAE* = 142.2272 kJ·mol^–1^, *MAPE* = 0.0484, and *RMSE* = 342.0464 kJ·mol^–1^, and *RMSLE* = 0.1172. Moreover, specific models were developed for different families of compounds, achieving *R* ^2^ ≈ 0.99 in all cases. Finally, a clustering analysis using the K-Means algorithm in the space defined by the topological indices enabled the identification of latent molecular patterns, providing a novel framework for organizing and analyzing chemical space. This work demonstrates the potential of combining supervised and unsupervised learning methods with chemical graph theory to enable accurate, robust, and scalable prediction of thermochemical properties such as combustion enthalpy.

## Graphical abstract

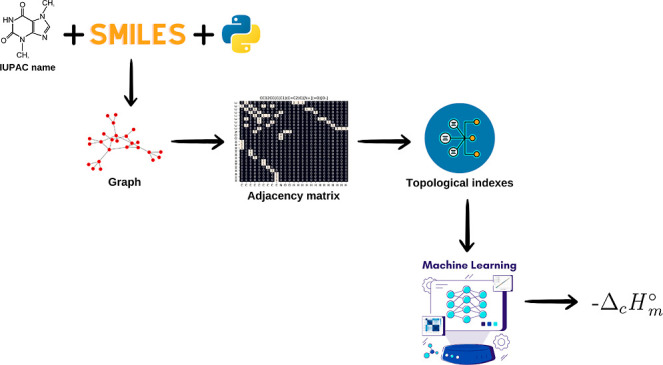

## Introduction

The enthalpy of combustion is a fundamental thermodynamic property that reflects the amount of energy released when a substance undergoes complete oxidation. This property is crucial for evaluating the energetic performance of organic compounds in various industrial and environmental applications. Traditionally, the experimental determination of combustion enthalpy is complex, costly, and requires stringent conditions, motivating the development of predictive methods based on computational models.^1–4^

Given the vast amount of information involved in modeling chemical reaction mechanisms, determining molecular conformations, and understanding thermodynamic properties, efficient data processing and organization are essential.^5–9^ Representing information as networks or graphs offers multiple perspectives, simplifies the understanding of phenomena, and helps address complex problems. Graphs employ descriptive statistics such as degree, closeness centrality, betweenness centrality, degree centrality, information centrality, and eigenvector centrality, which enable the analysis of the structure of nodes and edges.^10^

A prominent field that has emerged from the application of graph theory to chemical phenomena is chemical graph theory, which provides a framework to describe how molecular interactions influence the macroscopic properties of a system.^11–13^ Moreover, there are approaches that relate molecular structure to physical, chemical, biological, nanostructural, and toxicological properties, transforming the search for compounds with desired properties into predictive mathematical models based on quantified and computerized molecular descriptors. Among these methods are quantitative structure–property relationships (QSPR), quantitative structure–activity relationships (QSAR), quantitative nanostructure–activity relationships (QNAR), and quantitative structure–toxicity relationships (QSTR).^14–18^

Tools such as SMILES, BigSMILES, QM9, InChI, and SMARTS have facilitated access to relevant structural and molecular information, including molecular mass, adjacent matrices, Log *P*, and three-dimensional representations.^2,13,19,20^ Combining these techniques and tools has enabled the development of algorithms and workflows that use functional group contribution methods to estimate heat capacities, standard enthalpies of formation, and standard enthalpies of combustion.^1,2,21–28^

The experimental techniques to determine these values can be complex, especially given the high level of purity of the compound needed (∼99.99%), as well as the amount of sample required for each experiment and, if it is a recently synthesized product, the work increases exponentially.^29,30^ The application of graph theory to the prediction of standard molar enthalpies allows chemical graphs to describe how molecular components (atoms or functional groups) affect energetic properties.^31,32^

All of this has created the need for new strategies to obtain reliable estimates of target thermochemical properties by leveraging pre-existing theoretical, computational, and experimental information. One such innovative strategy is the implementation of machine learning (ML) models trained on carefully selected predictor variables, combining data from databases, molecular representations, and experimental measurements.^8,13,33,34^

Emerging strategies that combine ML with chemical graph theory and related data sources have proven effective for predicting thermochemical properties such as the standard enthalpy of combustion, using topological and statistical descriptors.^18,35^ In this work, such an approach was employed to successfully predict the standard combustion enthalpies of organic compounds, contributing to closing existing gaps in the precise and generalizable modeling of these properties.

### QSPR-Based Modeling of Combustion Enthalpy

The standard enthalpy of combustion is defined as the enthalpy change when one mole of a substance is completely burned in the presence of oxygen under standard conditions (1 bar). In other words, it represents the heat released during the combustion process of a substance, yielding products in their standard stable states (e.g., CO_2_(*g*), H_2_O­(l), and N_2_(*g*)). In general, combustion enthalpies are negative, indicating an exothermic process.^36^

The general combustion reaction for an organic compound with empirical formula C_*a*_H_*b*_O_*c*_N_*d*_ can be expressed as

1 $$C_{a}H_{b}O_{c}N_{d}+\left(a+\frac{b}{4}-\frac{c}{2}\right)O_{2}\to a{CO}_{2}+\frac{b}{2}H_{2}O+\frac{d}{2}N_{2}$$

Additionally, the enthalpy of combustion of a substance can be indirectly obtained if the standard enthalpies of formation of all reactants and products are known by applying Hess’s law. The enthalpy of reaction is calculated as

2 $$\Delta_{comb}H^{{}^{\circ}}=\sum_{i}\nu_{i}\Delta_{f}H^{{}^{\circ}}\left(products\right)-\sum_{j}\nu_{j}\Delta_{f}H^{{}^{\circ}}\left(reactants\right)$$

where ν_*i*_ and ν_*j*_ are the stoichiometric coefficients of the products and reactants, respectively, and *Δ_f_H°* denotes the standard enthalpy of formation of each species.^36^ This underscores the importance of accessing reliable and precise enthalpy of formation data, which are essential for accurately estimating the combustion enthalpy.

### Estimating Enthalpies of Formation: Group Contribution and Quantum Methods

Commonly, at the experimental level, data on standard enthalpies of formation are not always available for compounds of interest, which hinders the calculation of combustion enthalpy. Nevertheless, estimation methods have been developed, such as the one proposed by Benson.^37^ This method generally assumes that the enthalpy of formation of an organic compound can be obtained as a linear combination of the contributions from each functional group or structural fragment present in the molecule. That is, each group is assigned a specific enthalpy value, derived from experimental data of simple molecules. Therefore, by summing the contributions of all the groups, along with corrections for isomerism, it is possible to estimate the standard enthalpy of formation of the target molecule and, hence, its combustion enthalpy.^37^ In addition, modern variants have been developed that include atomic or bond-fragment contributions and even methodologies that derive group contributions through ML techniques.^38,39^

Beyond group contribution methods and their modern alternatives, standard enthalpies of formation can also be obtained from quantum chemical methods.^40^ Ab initio and density functional theory (DFT) methods have been employed to compute absolute electronic energies.^41^ By combining these energies for the reactants and products in formation reactions, the enthalpy of formation of the compound can be derived. Recent studies have employed high-precision quantum methods such as G4 and CBS-QB3 to predict formation enthalpies with uncertainties of only a few kJ/mol.^42–44^ For instance, formation enthalpies of explosive molecules have been calculated using DFT, yielding results close to experimental values.^45,46^ Likewise, other studies have reported accurate formation enthalpies using atomic equivalent energy approaches derived from semiempirical DFT (DFTB) calculations.^47,48^ These theory-based approaches are useful for novel or uncommon compounds, where group contribution methods may lack reported values for new functional or structural groups. However, they are computationally expensive and limited to the gaseous phase.

### Topological Indices of Graphs in QSPR

In parallel with the aforementioned methods, mathematical chemistry and cheminformatics have developed approaches to derive molecular descriptors that capture the structure of chemical compounds.^49,50^ In particular, chemical graph theory is a useful tool for representing molecules as undirected graphs, where atoms are defined as nodes and chemical bonds as edges.^51^ Using statistical and linear algebra tools, it is possible to extract topological characteristics of molecular graphs.^52^ A topological index is essentially described as a numerical topological property of the molecular graph that remains invariant under isomorphisms. This enables the establishment of topology–physicochemical property relationships, that is, QSPR relationships.^53^

Consequently, these topological indices can be used as molecular descriptors to predict a wide variety of physicochemical properties or to predict molecular activity.^52^ Currently, there is a large variety of topological indices, which can be essentially classified into three types:^52,54^

1. Degree-based indices, which depend solely on the number of bonds each atom has.^55^ Among the most commonly used are the Zagreb indices and the Randić index, which have been studied in relation to molecular branching and correlations with properties such as boiling points of alkanes, respectively.^56,57^ Other popular indices include the atom–bond connectivity (ABC) index^58^ and the geometric–arithmetic (GA) index,^59^ both of which have been related to the energetic stability of hydrocarbons.
2. Distance-based indices, which are derived from the distances between pairs of nodes or atoms within a molecule. The most prominent among these is the Wiener index, which has shown correlations with the boiling points of alkanes.^60,61^ Another example is the Gutman index, which is obtained by weighting the distances between atoms using their degrees.^62^
3. Adjacency spectral indices, which are calculated from the eigenvalues of the adjacency matrix of the molecular graph.^63^ A notable index in this category is the Estrada index, which captures global connectivity information on the molecular graph, including subtle effects of the distribution of single and multiple bonds.^64^ It has been shown to be applicable in the study of π-electronic energies in aromatic hydrocarbons as well as in the prediction of pharmacological properties in QSAR studies.^65^

In recent scientific literature, numerous studies have reported the development of QSPR models aimed at predicting physicochemical properties of various types of organic compounds using topological indices of the three main categories previously described. For instance, a study employing 16 different distance-based indices—such as Wiener, hyper-Wiener, and Harary indices—demonstrated their utility in predicting properties like boiling point, enthalpy of vaporization, and log *P* for a data set of 19 antiasthmatic drugs. In all cases, the models achieved *R* ^2^ values greater than 0.9; however, only linear regression models were employed.^66^ Similarly, the ZEP index (based on degree and electronic distances) was used to predict the polarizability of a data set comprising 84 aliphatic carboxylic acids, achieving *R* ^2^ values above 0.99 for training, leave-one-out cross-validation, and testing stages, again using linear regression.^67^ In addition, a recent study compared six versions of the Sombor index for the prediction of flash point in 38 polycyclic aromatic hydrocarbons, employing both linear and second- and third-order polynomial models, which yielded *R* ^2^ values exceeding 0.94.^68^

Regarding degree-based indices, several classical descriptors such as the ABC index, first- and second-order Zagreb indices, Randić index, harmonic index, and sum-connectivity index have been employed to model physicochemical properties such as flash point, enthalpy of formation, and molar refractivity in a data set of 13 antipsychotic drugs. These models reported correlation coefficients greater than 0.9 using linear regressions.^69^ Likewise, eccentricity-based indices (e.g., NE, NE, NFE, NRE) have been applied to predict properties including boiling point, enthalpy of vaporization, flash point, molar refractivity, and polarizability in 8 antiviral drugs, achieving *R* ^2^ values above 0.9 with linear models and close to 0.99 using cubic models.^70^ Moreover, the Neighborhood Face Index (NFI) was employed to predict -electron energy and other molecular properties for a set of 21 benzenoid compounds using linear regression models, yielding correlation values near 0.99.^71^ Variants of the Zagreb and harmonic indices have also been used to model molar volume and enthalpy of vaporization in a data set of 16 alkaloid compounds, with reported *R* ^2^ values exceeding 0.9 in linear models.^72^ Furthermore, the application of generalized multiplicative Zagreb indices has been extended to the prediction of enthalpy of formation in 25 aromatic hydrocarbons, achieving *R* ^2^ values as high as 0.97 using linear regression.^73^

Although there has been substantial progress in the use of degree- and distance-based topological indices for QSPR modeling of physicochemical properties, relatively few studies have systematically explored the application of pure spectral indices in this context. For example, the prediction of properties such as molar volume, polarizability, and molar refractivity has been investigated for 8 pharmaceutical compounds using graph energy-based descriptors, such as adjacency energy, within linear models, obtaining correlation coefficient values greater than 0.98. Additionally, spectral indices have been explored for modeling entropy and heat capacity in a data set of 30 polycyclic hydrocarbons, with correlation coefficients exceeding 0.95.^74^

These findings suggest that the use of spectral indices remains an emerging area within QSPR modeling, offering significant potential for future studies that incorporate supervised and unsupervised learning techniques, as well as applications to more chemically diverse data sets.

### QSPR Models Based on Machine Learning

Traditionally, QSPR models for physicochemical properties such as enthalpy were built using linear regression models with only a few descriptors, such as the number of carbon, hydrogen, oxygen, and nitrogen atoms, among others. However, many physicochemical properties can exhibit complex nonlinear relationships with molecular structure, hence the need to use ML models.^75,76^ Unlike conventional QSPR models, ML-based models can uncover nonlinear patterns and capture high-dimensional relationships within the data. Examples of such techniques include k-nearest neighbors, support vector machines, decision trees, and, of course, the widely known neural networks.^77,78^

Although these models have demonstrated strong predictive power, most of them lack interpretability, making it difficult to understand how a set of independent variables contributes to predicting a dependent variable and to what extent.^79,80^ Several studies have reported the use of ML for the prediction of thermochemical properties. For example, artificial neural networks have been applied to predict the combustion enthalpy of oxygenated fuels with 96.3% accuracy. This model used 14 functional group contributions as predictive variables.^81^ However, the major drawback remains interpretability: how much and why each descriptor contributed to the enthalpy prediction.

When building QSPR models using ML techniques for regression problems, it is essential to rigorously assess their performance. For this reason, multiple error metrics are typically used, among which the most prominent include:

(1) Mean Absolute Error (*MAE*): indicates the average deviation of predictions from actual values.

3 $$MAE=\frac{1}{n}\sum_{i=1}^{n}\left|y_{i}-{ŷ}_{i}\right|$$

where *n* is the total number of observations, *y*_*i*_ is the actual value of the *i*th observation, and ${ŷ}_{i}$ is the predicted value for that observation. This metric is expressed in the same units as the target property and is easy to interpret.^82^

(2) Root mean squared error (*RMSE*): penalizes large errors more than MAE by squaring the deviations before averaging.

4 $$\mathrm{RMSE}=\sqrt{\frac{1}{n}\sum_{i=1}^{n}{\left(y_{i}-{ŷ}_{i}\right)}^{2}}$$

(3) Mean absolute percentage error (*MAPE*): represents the error as a percentage of the true value, making it intuitive for communicating results.^82^

$$MAPE=\frac{100}{n}\overset{n}{\underset{i=1}{\sum }}\left|\frac{y_{i}-{ŷ}_{i}}{y_{i}}\right|$$ 5

(4) Root mean squared logarithmic error (*RMSLE*): similar to *RMSE*, but penalizes errors proportionally on a logarithmic scale. It is especially useful when modeling properties that vary across orders of magnitude, as it reduces the influence of large outliers.^82^

$$RMSLE=\sqrt{\frac{1}{n}\overset{n}{\underset{i=1}{\sum }}{\left(\log\left({ŷ}_{i}+1\right)-\log\left(y_{i}+1\right)\right)}^{2}}$$ 6

(5) Coefficient of determination (*R* ^2^): although not a true error metric, this is one of the most commonly used metrics to evaluate regression models. It represents the proportion of variance in the dependent variable that is explained by the model.

7 $$R^{2}=1-\frac{\sum_{i=1}^{n}{\left(y_{i}-{ŷ}_{i}\right)}^{2}}{\sum_{i=1}^{n}{\left(y_{i}-\mathrm{y̅}\right)}^{2}}$$

where *y̅* is the mean of all observed values. A value of *R* ^2^ close to (1) suggests a good model fit. However, a high *R* ^2^ alone does not ensure generalizability, so it should be complemented with other error metrics.^82^

### Evaluation Strategies in ML-Based QSPR Modeling

Within the training, evaluation, and testing of ML models for QSPR, not only do evaluation metrics matter, but also the evaluation methods or strategies, as model performance largely depends on the chosen strategy.^83,84^ It is well-known that it is poor practice to train ML models using the entire available data set and then report their performance on the same data, as this can lead to underfitting or overfitting.^85,86^

There are several evaluation methods, such as the hold-out method. This method involves partitioning the total available data set into two subsets: one for training and the other for evaluating real predictive performance. Typical splits include 70/30, 80/20, or 90/10. This approach is simple but ensures that the generalization error can be quantified using unseen data during training. This method is particularly useful when large data sets are available.^84^

Another common method is k-fold cross-validation (k-fold CV). Instead of generating a single split, the complete data set is divided into *k* subsets. The ML model is trained *k* times using *k* – 1 folds, while the remaining fold is used for validation. This procedure is repeated by rotating the folds so that each sample is predicted exactly once when it is part of the validation fold.^87^ Finally, the evaluation metrics obtained from the *k* folds are averaged. This method provides a more robust and stable evaluation mechanism for the selected ML model. It is also used to tune the hyperparameters of an ML model and helps prevent overfitting.

There are variations of this technique, such as stratified cross-validation for classification problems, leave-one-out cross-validation (LOO), and nested cross-validation, among others.^87^ Nested cross-validation is highly recommended when fine-tuning hyperparameters or comparing models fairly.^88,89^ In general terms, it involves nesting one cross-validation routine within another. For example, in an outer 5-fold CV, 4/5 of the data is used for model optimization, i.e., internal training and validation and 1/5 is used for testing. Within the 4/5 training subset, an inner CV is performed to select the best hyperparameters or predictive variables. Thus, the optimization process occurs only on internal training data, and the external evaluation is performed with data that was never used for either training or hyperparameter selection, resulting in a more realistic estimation of model performance. This technique allows for precise model tuning and yields an unbiased estimate that avoids overfitting due to hyperparameter selection. However, it is computationally expensive.

### Random Forest and Extra Trees Algorithms in QSPR

Among the currently available ML models, ensemble models based on decision trees have gained popularity in QSPR due to their ability to capture nonlinear structure–property relationships, their robustness against overfitting, and a favorable balance between predictive power and interpretability. Two prominent ensemble methods are Random forest (RF) and Extremely randomized trees (ET).^90–92^

The RF algorithm constructs an ensemble of *T* decision trees, each trained on a random subset of the data and descriptors, and combines their predictions by averaging. The prediction for a molecule described by a feature vector *x* is given by

8 $${ŷ}_{RF}\left(x\right)=\frac{1}{T}\sum_{t=1}^{T}h_{t}\left(x\right)$$

where *h*_*t*_(*x*) is the prediction of the *t*th tree.

Each tree is built using a process known as bootstrap aggregating (bagging), where a random sample with replacement from the training set is used. Furthermore, at each node of a tree, only a random subset of molecular descriptors is considered to find the optimal split. This randomness promotes diversity among the trees, reducing prediction variance and improving generalization.

For regression tasks such as combustion enthalpy prediction, each node seeks the split that minimizes the weighted Mean Squared Error (*MSE*) (or another evaluation metric previously described) of the child nodes

$$\underset{j,s}{\min}\left[\frac{\left|S_{L}\right|}{\left|S\right|}\cdot MSE\left(S_{L}\right)+\frac{\left|S_{R}\right|}{\left|S\right|}\cdot MSE\left(S_{R}\right)\right]$$ 9

where *j* indexes the candidate predictors (e.g., molecular or topological descriptors), and *s* denotes a threshold value used to split the data set *S* into two subsets: *S*_L_ and *S*_R_, corresponding to the left and right branches, respectively. The goal is to find the pair (*j*, *s*) that yields the best reduction in impurity.

The *MSE* for a subset *S* is defined as

$$MSE\left(S\right)=\frac{1}{\left|S\right|}\underset{i\in S}{\sum }{\left(y_{i}-{\mathrm{y̅}}_{S}\right)}^{2}$$ 10

with *y*_*i*_ as the actual target value of sample *i*, and ${\mathrm{y̅}}_{S}$ as the mean target value in subset S.

This recursive splitting continues until stopping criteria are met (e.g., a minimum number of samples per leaf), and each leaf then outputs the mean of the target values in its region.^90,91^

The ET algorithm builds upon the RF idea but introduces additional randomness. While RF searches for the best split threshold among candidate features, ET selects thresholds randomly for each feature and chooses the one yielding the best performance. Specifically, for each node: a random subset of *K* candidate features *x*_*j*_ is selected; then, for each *x*_*j*_, a threshold θ is randomly drawn within its value range in that node; finally the best among these random splits (e.g., lowest *MSE*) is chosen.

Unlike RF, the ET model typically uses the entire training data set for each tree (no bootstrap sampling) but still applies random splits. This results in trees that are more diverse, further reducing overfitting when predictions are averaged although more trees may be required to stabilize the ensemble prediction.^93,94^

In summary, both RF and ET rely on the same averaging strategy for prediction. The key difference lies in how splits are generated: RF searches for optimal thresholds among a subset of features, whereas ET introduces randomness in both feature and threshold selection. Because of their robustness, ability to handle high-dimensional data, and compatibility with interpretability tools such as feature importance analysis and SHAP (SHapley Additive exPlanations) method,^95,96^ these algorithms are well-suited for developing QSPR models of thermochemical properties like combustion enthalpy.

### Interpretability and Explainable AI

One advantage of these ensemble models compared to other ML approaches in their application to QSPR is their relative interpretability and their contribution to explainable artificial intelligence (XAI).^97,98^ Although they are considered black-box models in a strict sense, they offer internal mechanisms to assess the importance of predictive variables. This allows for the identification of the most influential factors, for example, in the prediction of combustion enthalpy or other physicochemical properties.

Among the most commonly used tools to improve the interpretability of these models are partial dependence plots (PDPs), which show how the average prediction of the model varies when a single predictor is modified while averaging over the others.^99^ This visualization helps detect nonlinear relationships between descriptors and the target property.

Furthermore, it is possible to examine individual trees within the ensemble. A notable technique is CHIRPS (Collection of High Importance Random Path Snippets), which allows for the extraction of highly interpretable subtrees from the forest, revealing specific decision paths that contribute significantly to the model’s predictions.^100^

In addition, game theory-based techniques such as SHAP can be employed. These decompose the prediction of a model into additive contributions attributable to each independent variable. SHAP provides both local and global measures of feature importance, offering detailed insight into how each descriptor influences the final prediction.^95,96^ This is particularly valuable in nonparametric models, such as decision tree ensembles, that capture highly complex and nonlinear relationships.

Currently, research on the use of ML models for building QSPR models is very active and has produced promising results. However, several knowledge gaps and challenges remain in this field. First, many QSPR models for predicting formation or combustion enthalpies have been developed using data sets composed of relatively homogeneous chemical families, which facilitates finding internal correlations but limits generalizability.^66–73^ Therefore, one of the main challenges is to expand the applicability of these models to structurally diverse compounds.

Another major challenge is the trade-off between interpretability and predictive performance. The most accurate models, such as deep neural networks, are often difficult to interpret, hindering the extraction of underlying chemical principles.^79,80^ If a model is not interpretable, it becomes difficult to determine whether it captures a chemically valid pattern or merely spurious correlations. Hence, the importance of incorporating XAI techniques into the development of QSPR models, a strategy that has not yet been widely explored in the context of thermochemical property prediction.

## Computational Details

Workflow for this work followed the next stages: obtaining structural information on organic compounds, construction of data set, preprocessing the data set, extracting molecular descriptors of the organic compounds along with statistical properties and topological indexes of these molecules, performing exploratory data analysis, and applying supervised and unsupervised machine learning models (see Figure 1).

**Figure 1:**
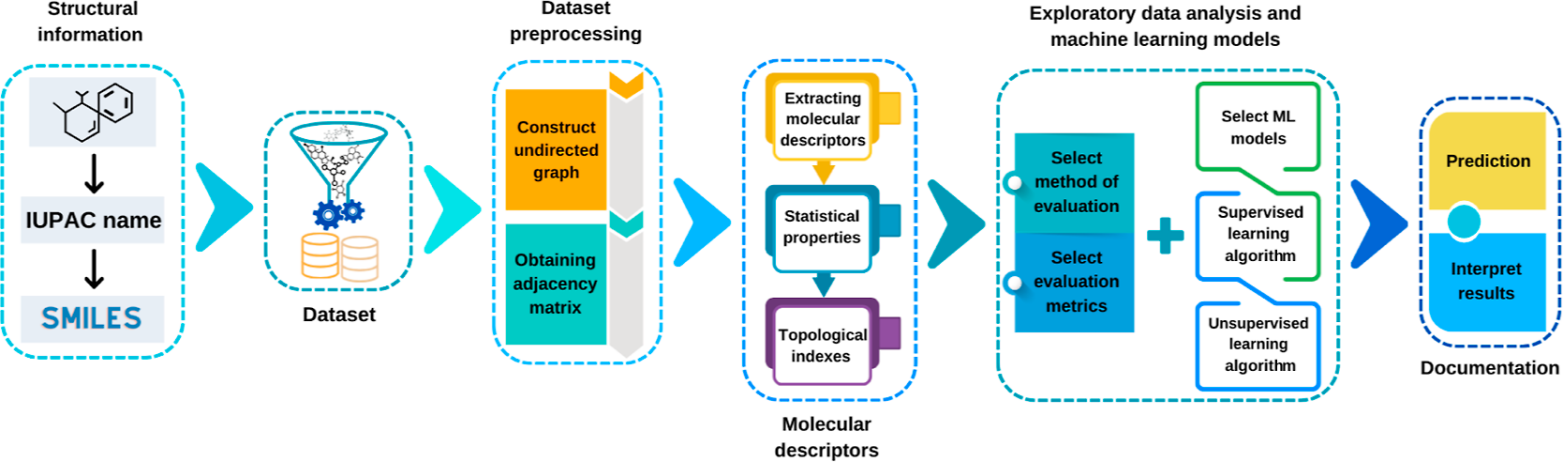
Schematic representation of the workflow used for predicting the standard enthalpy of combustion of 3477 organic compounds. Structural data (IUPAC names and SMILES) are obtained from the PubChem platform, the data set is constructed and preprocessed to create the molecular graph and adjacency matrix, molecular descriptors (statistical, topological, and molecular indices) are extracted, exploratory data analysis is carried out to find correlations, models are trained using supervised and unsupervised learning algorithms, and the results are interpreted.

### Structural Information of Organic Compounds

As previously stated, a data set comprising 3477 organic compounds was used. This data set contains the IUPAC names for the compounds, their state of matter (solid, liquid, or gas), and their standard combustion enthalpy values.^101^ The programming language used as the computational tool for data analysis was Python V3.13 (https://www.python.org/).

First, the PubChemPy library V1.0.4 (https://pubchempy.readthedocs.io/en/latest/guide/introduction.html) for Python was used to retrieve the IUPAC names of each of the 3477 compounds from the PubChem database (https://pubchem.ncbi.nlm.nih.gov). This library allows searches in the database using various identifiers, such as IUPAC names, compact chemical formulas, InChI, SMILES, or InChIKey. In this study, we used the IUPAC names of the molecules to obtain their SMILES (Simplified Molecular Input Line Entry System) identifiers, which provide a linear and simplified representation of a molecule’s chemical structure.

For context, SMILES is a text-based format that describes how atoms in a molecule are connected and includes specific symbols for bonds, cycles, and branches.^19^ This identifier is widely used in computational chemistry, bioinformatics, chemoinformatics, and molecular modeling because it enables compact and readable descriptions of molecules, facilitating interpretation by computers.^102–104^

### Data Preprocessing

SMILES codes obtained with PubChemPy were read by the Python libraries PySmiles v1.1.2 (https://github.com/pckroon/pysmiles) and NetworkX v3.4.2 (https://networkx.org)^105^ which constructed the undirected graph: *G* = (*V*, *E*) where *V* is the set of nodes (vertices) for the graph, that is to say, every atom in each molecule, and *E* is the set of edges between pairs of nodes within the graph, namely, the bonds between atoms in a molecule. For an undirected graph, the edges are described as

11 $$E\subseteq \left\{\left\{v_{i},v_{j}\right\}|v_{i},v_{j}\in Vyi\neq j\right\}$$

where *E* is the set of edges, *V* is the set of vertices, *v*_*i*_ and *v*_*j*_ are vertices of the graph (*i* ≠ *j* indicates that there are no loops an edge does not connect a vertex to itself). Each graph has an associated adjacency matrix.^10^ The adjacency matrix *A* is a matrix of size *nxn* defined as

12 $$A\left[i\right]\left[j\right]=\{\begin{array}{ll} 1 & \text{if}\;there\;is\;an\;edge\;between\;v_{i}\;\text{and}\;v_{j},\\ 0 & otherwise. \end{array}$$

The process of constructing a graph and its respective adjacency matrix from a molecule’s IUPAC name is summarized in Figure 2.

**Figure 2:**
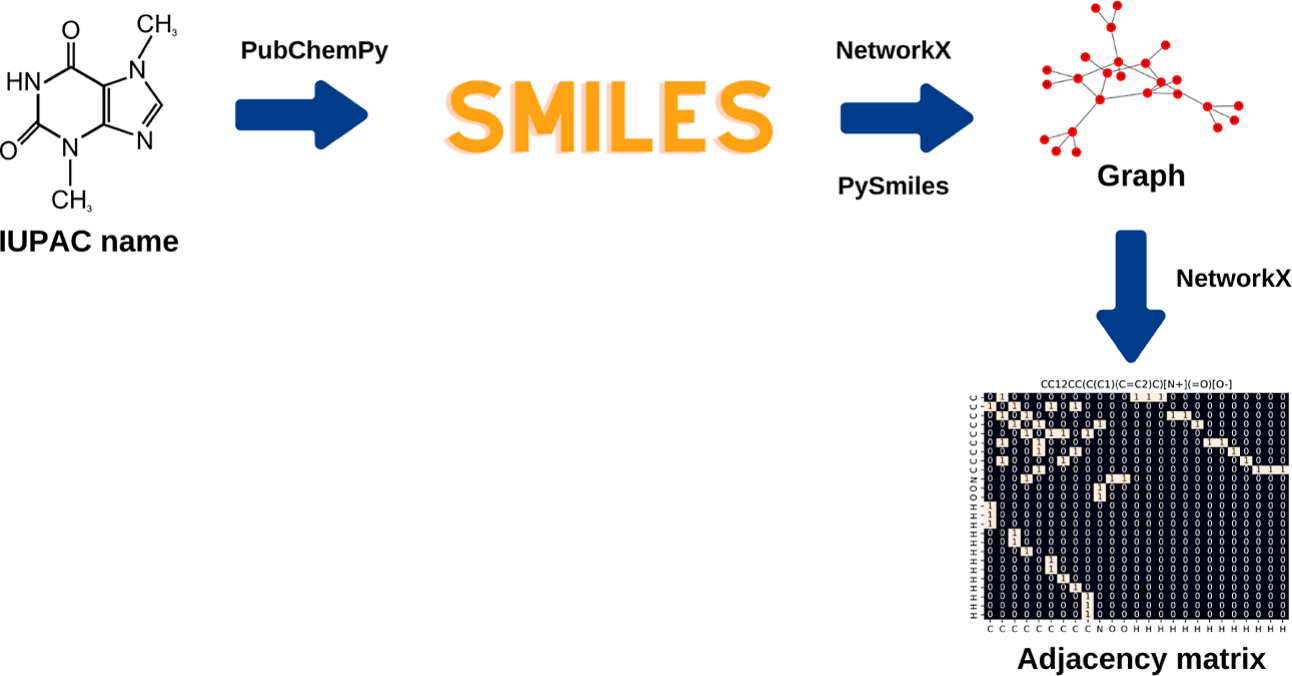
Procedure for constructing the graphs of each organic compound, as well as the corresponding adjacency matrix, from the IUPAC name obtained via PubChem. The workflow involves retrieving the SMILES notation of each compound using the PubChemPy library, converting it into a molecular graph using the PySMILES and NetworkX libraries, and computing the adjacency matrix that represents atomic connectivity.

### Molecular Descriptors and Topological Indices

After obtaining the graphs, the following statistical properties were calculated using the NetworkX v3.4.2 library: Degree,^106^ Betweenness Centrality,^107^ Closeness Centrality,^108^ and eigenvector Centrality,^109^ as well as the topological indices of Estrada,^110^ Gutman,^111^ and Wiener.^112^

Additionally, 210 molecular descriptors were employed, all of which were obtained using RDKit (https://www.rdkit.org/docs/api-docs.html). According to their dimensionality, these descriptors can be categorized. A total of 31 one-dimensional (1D) descriptors were included, which infer compound properties solely based on their chemical formula, without considering distances or connectivity beyond direct neighbors, i.e., they rely on information related to individual nodes. Furthermore, 140 two-dimensional (2D) descriptors were used; these incorporate information derived from the compound’s two-dimensional topological structure, considering distances or paths between nodes within the molecular graph. In addition, 39 three-dimensional (3D) descriptors were included, which account for the spatial shape of the compound in a specific conformation or for structural aspects associated with graph geometry and energy distribution.^113–115^ A detailed description of each descriptor obtained from RDKit is provided in Table S1.

### Statistical Properties and Topological Indices of Graphs

In order to predict standard enthalpy of formation values, we employed various graph-theoretical descriptors that capture molecular topology. These include centrality measures and topological indices derived from the molecular graph’s structure.

The degree of a node, defined as the number of edges incident to it, reflects its direct connectivity. It is expressed as deg­(*v*) = ∑_u_A_vu_, where A_vu_ is the element of the adjacency matrix *A* corresponding to the connection between nodes *v* and *u*.^106^

Betweenness centrality quantifies the extent to which a node lies on the shortest paths between other nodes. It is calculated as $C_{B}\left(v\right)=\sum_{s\neq v\neq t}\frac{\sigma_{st}\left(v\right)}{\sigma_{st}}$ , where σ_st_ is the total number of shortest paths from node *s* to node *t*, and σ_st_(v) is the number of those paths that pass through node *v*.^107^

Closeness centrality evaluates how close a node is to all other nodes in the graph. It is defined as $C_{C}\left(v\right)=\frac{1}{\sum_{t\neq v}d\left(v,t\right)}$ , where *d*(*v*, *t*) denotes the shortest path between nodes *v* and *t*.^108^

Eigenvector centrality assigns a score to each node based on the principle that connections to high-scoring nodes contribute more to the score. It satisfies the equation $C_{E}\left(v\right)=\frac{1}{\lambda }\sum_{u}A_{vu}C_{E}\left(u\right)$ , where λ is the largest eigenvalue of the adjacency matrix *A*.^109^

The Estrada index provides a measure of the overall subgraph centrality and vibrational energy of a graph. It is computed as $EE=\sum_{i=1}^{n}e^{\lambda_{i}}$ , where λ_*i*_ are the eigenvalues of the adjacency matrix *A*.^110^

The Gutman index incorporates both vertex degrees and topological distances. It is defined as Gutman­(*G*) = ∑_*i*<*j*_ d­(*i*, *j*)·deg­(*i*)·deg­(*j*), where d­(*i*, *j*) is the shortest path distance between nodes *i* and *j*, and deg­(*i*) is the degree of node *i*.^111^

Finally, the Wiener index measures the compactness of a graph by summing the shortest path distances between all pairs of vertices: *W*(*G*) = ∑_*i*<*j*_ d­(*i*, *j*).^112^

### Exploratory Data Analysis and Application of Machine Learning Models

Once the statistical properties of the representative graphs for each molecule and their corresponding topological indices were obtained, 19 supervised regression models were trained, evaluated, and tested in order to identify the best estimator for the standard combustion enthalpy, as well as the most relevant predictor variables.^116^ The predictor variables were scaled using standard normalization or z-score scaling (zero mean and unit standard deviation). To achieve this, the data set was divided into two subsets: the first comprised 70% of the total data and was used to train and validate the estimators using 10-fold cross-validation, while the remaining 30% was used for testing.^89,117–120^ The evaluation metrics were *R* ^2^, *MAE*, *MAPE*, *RMSE*, and *RMSLE*.^117^ In this work, *MAPE* and *RMSLE* were added as evaluation metrics because the standard combustion enthalpy can take on small or extremely large values due to the variety of organic compounds in the data set. In cases like this, measuring errors on a relative scale becomes especially important to avoid unfairly penalizing predictions in one range or another. In this sense, *MAPE* focuses on the percentage error relative to the true value, so that a difference of 10 units for a real value of 1000 is very different from a difference of 10 units for a real value of 100. In other words, *MAPE* assesses the proportionality of the error without letting larger values overshadow smaller ones or vice versa.

In contrast, *RMSLE* applies a logarithm to both the predicted and actual values before calculating the mean squared error, reducing the influence of very large values and placing greater importance on accurately predicting smaller quantities. This logarithmic transformation ensures that a multiplicative error (for instance, predicting double or half of a value) is penalized more evenly across the entire spectrum of values, preventing errors on large values from completely dominating the metric. For this reason, although metrics such as *MAE* and *RMSE* can indicate that a model is overfitting, in data sets where the target variable(s) can span different orders of magnitude, it is advisible to compute *MAPE* and *RMSLE* to verify the presence of overfitting in ML models.

After identifying the estimator with the best evaluation metrics and the most important predictor variables, a random grid search with 5-fold cross-validation was performed to tune the model’s hyperparameters,^121^ obtain its learning and validation curves, analyze residuals on the training and testing sets, and generate parity plots for each subset.^117^

To compare the results obtained using the statistical and topological properties of the representative graphs of organic molecules as predictor variables, the same 19 supervised regression learning models were retrained, validated, and tested using the 210 molecular descriptors obtained from RDKit as predictor variables for the standard combustion enthalpy. The same predictor variable scaling method, evaluation method, and metrics were applied. Then hyperparameter tuning of the selected estimator was carried out using a random grid search with 5-fold cross-validation. Learning and validation curves, as well as residual and parity plots for each data subset, were also generated.

Lastly, unsupervised learning models^117^ were used to identify clusters of compounds with similar characteristics using the standard combustion enthalpy and the Estrada, Wiener, and Gutman indices as clustering variables. To determine the best clustering of the data, three unsupervised learning models were applied: K-Means,^122,123^ DBSCAN,^124,125^ and HDBSCAN.^126,127^ The elbow method was used to identify the optimal number of clusters for each, and clustering quality was assessed using the silhouette coefficient^128^ and the Davies-Bouldin score.^129,130^

## Results and Discussion

Figure 3 shows the Pearson correlation matrix^131^ between the topological and statistical properties of molecular graphs and the standard enthalpy of combustion (*−*Δ_c_ *H°*). A strong positive correlation is observed between the Estrada, Wiener, and Gutman indices and the enthalpy, suggesting that these descriptors capture relevant structural information associated with thermochemical behavior. In contrast, betweenness centrality (BC) shows a moderate negative correlation, while closeness centrality (CC) and eigenvector centrality (EC) exhibit weak correlations.

**Figure 3:**
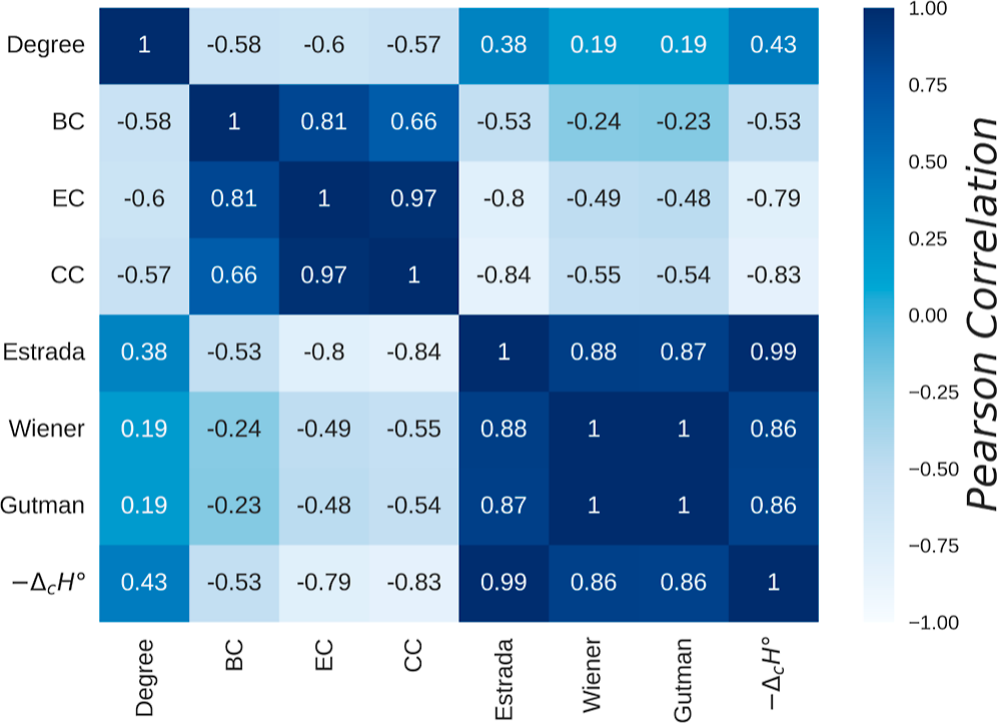
Pearson correlation matrix showing the degree of association between the statistical properties and topological indices of molecular graphs with the standard enthalpy of combustion (−Δ_c_ *H*°) of organic compounds.The matrix reveals a strong positive correlation between the Estrada, Wiener, and Gutman indices and the enthalpy of combustion, suggesting that these topological descriptors capture relevant structural information associated with thermochemical behavior. While Closeness (CC) and eigenvector (EC) centralities exhibit a moderate linear correlation with the enthalpy, it is comparatively lower. The degree and betweenness centrality (BC) exhibit weak correlations.

Among the descriptors, the Estrada index stands out due to its almost perfect linear correlation with combustion enthalpy (*R* ^2^ = 0.9734), as shown in Figure 4. The deviations from the regression line correspond to specific compound families, such as amines, although the general trend remains consistent across groups (see Figure S1). This index is derived from the spectral analysis of the adjacency matrix of the molecular graph *G*, thereby encoding 2D connectivity, but it is also indirectly influenced by 3D structure, since the arrangement of bonds and functional groups affects the spatial folding of the molecule.^110,132^ Additionally, through transformations such as the line graphs *L*(*G*), *L* ^2^(*G*), and *L* ^3^(*G*) whose nodes represent bonds, bond angles, and dihedral angles, respectively it is possible to generate a three-dimensional version of the index by weighting the adjacency matrix of *L* ^3^(*G*) with functions related to these angles.^110,132^

**Figure 4:**
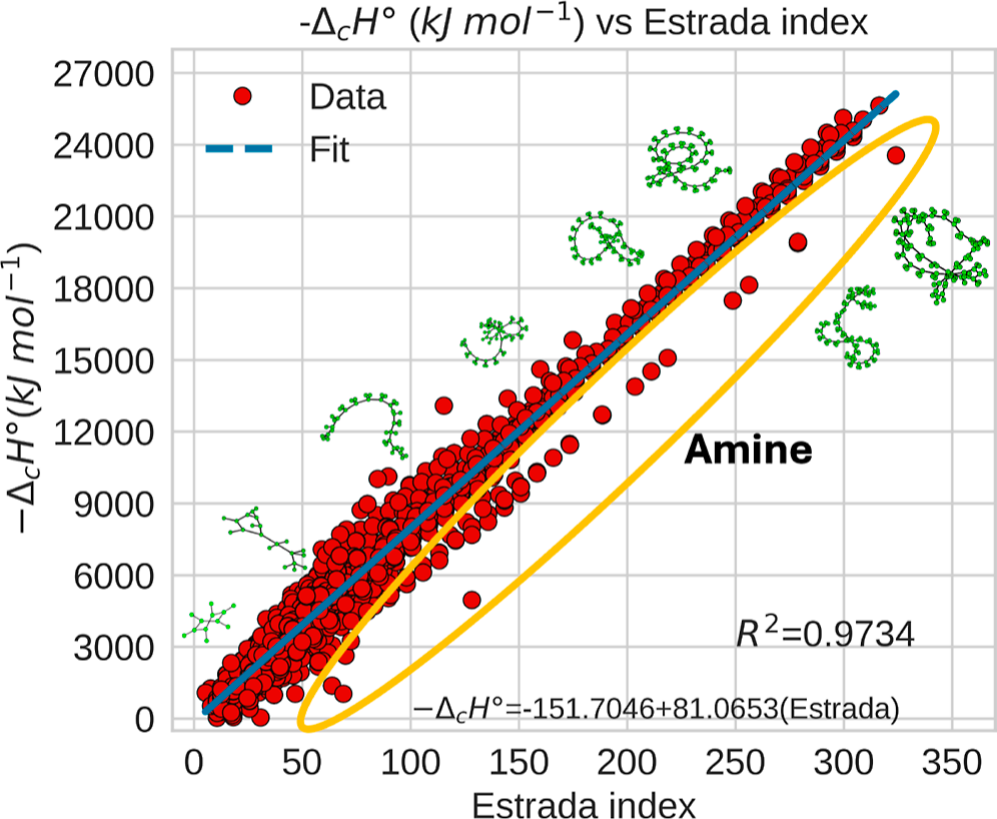
Standard combustion enthalpy as a function of the Estrada index. The image shows the graphs for benzene (28.7159), propylbenzene (51.2771), tetradecane (108.4246), heptadecylbenzene (156.7105), pentacosylbenzene (216.9582), nonacosylcyclopentane (256.1882), tritriacontylbenzene (277.2059), and pentatriacontylbenzene (292.2678). The number in parentheses is the value of the Estrada index.

Thermochemical properties, such as combustion enthalpy, are closely related to the three-dimensional structure of molecules,^10,133^ being influenced by factors such as bond types, bond energy, isomerism, the presence of alicyclic or aromatic structures, molecular size and complexity, and steric effects.^104,113^

In this context, other topological indices also reflect relevant structural features. The Wiener index, defined as the sum of the shortest distances between all pairs of atoms in a molecule,^112^ exhibits a nonlinear relationship with combustion enthalpy (*R* ^2^ = 0.9677), as shown in Figure 5. Although it is a two-dimensional descriptor, it provides insight into the compactness or branching of the molecule, characteristics that affect its spatial properties and thus its thermochemical behavior^104,134^ (see also Figure S2).

**Figure 5:**
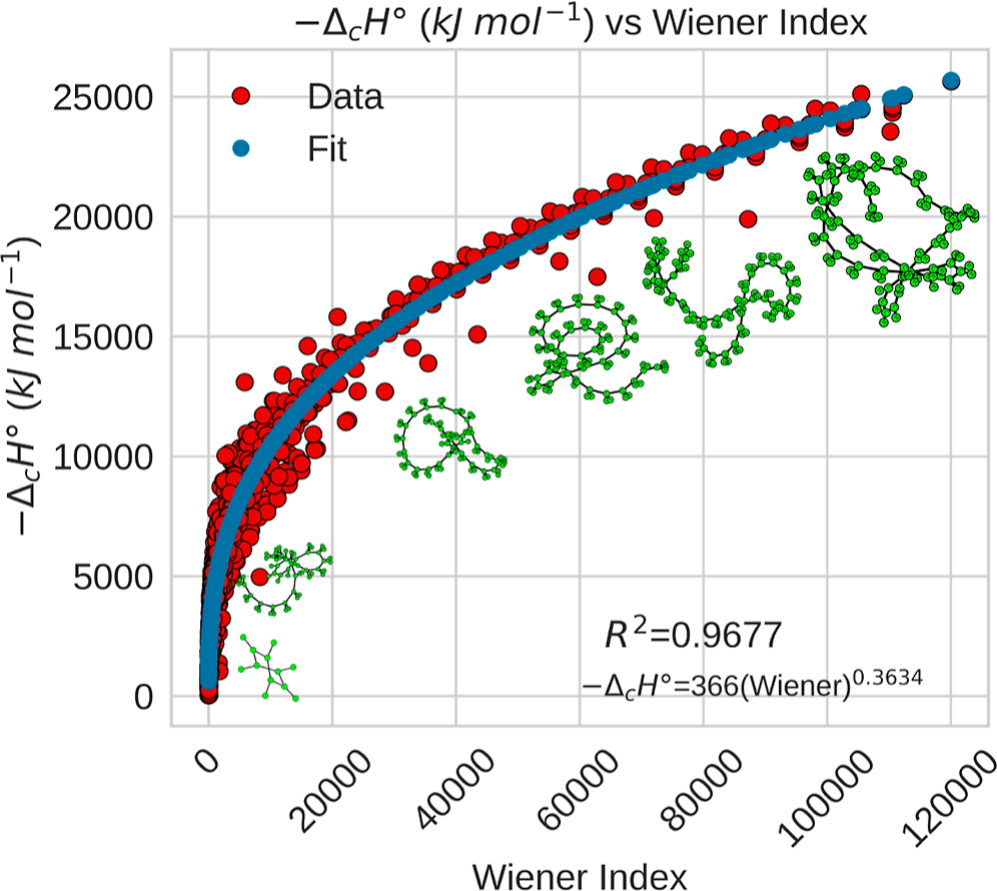
Standard combustion enthalpy as a function of the Wiener index. The image shows the graphs for benzene (570), heptadecylbenzene (16494), pentacosylbenzene (41574), nonacosylcyclopentane (65003), tritriacontylbenzene (84126), and pentatriacontylbenzene (98034).The number in parentheses is the value of the Wiener index. In a manner similar to the Estrada index, the points that deviate from the trend line can be attributed to specific groupings of organic compound families.

Similarly, the Gutman index (also known as the modified Zagreb index), based on the degree of the nodes in the molecular graph, has been widely used to study the stability, reactivity, and bond energy of compounds.^111,113^ As shown in Figure 6, this index also correlates nonlinearly with combustion enthalpy (*R* ^2^ = 0.9712), with a consistent trend across different compound families (see Figure S3). High Gutman index values are generally associated with densely connected and therefore more stable structures.^34,111,134^

**Figure 6:**
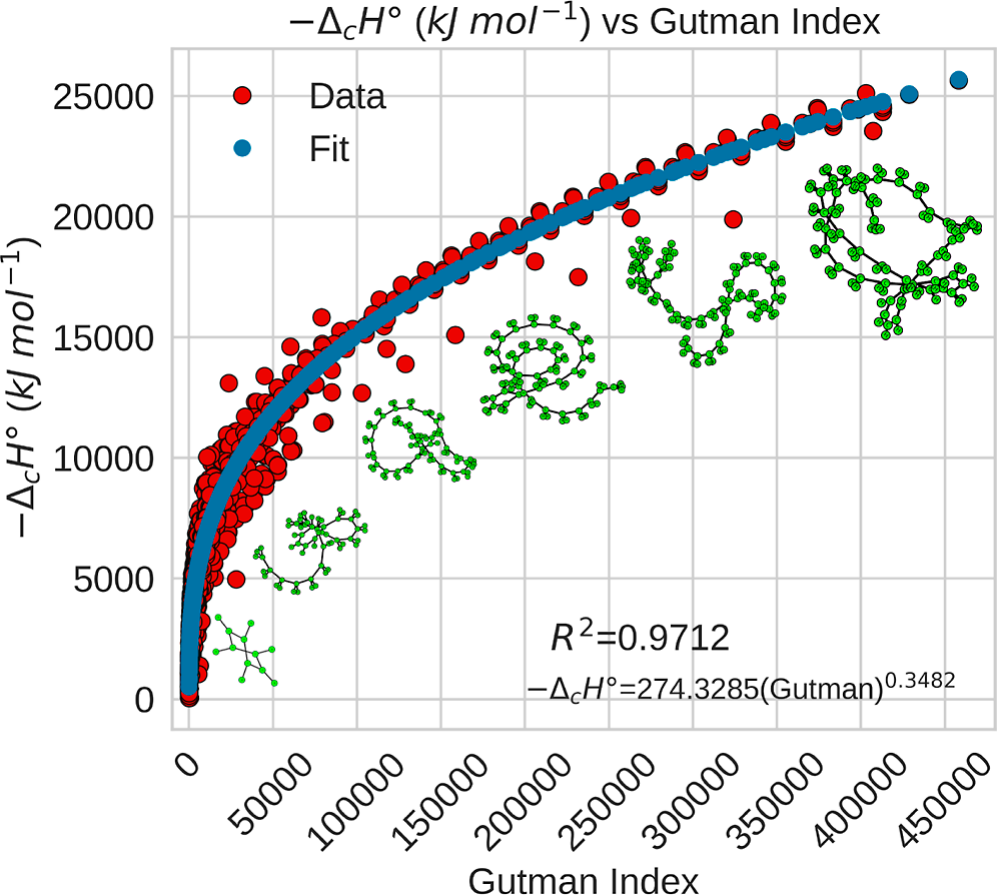
Standard combustion enthalpy as a function of the Gutman index. The image shows the graphs for benzene (570), heptadecylbenzene (60801), pentacosylbenzene (156345), nonacosylcyclopentane (245735), tritriacontylbenzene (320241), and pentatriacontylbenzene (374055).The number in parentheses is the value of the Gutman index.

By combining these three indices in a multiple linear regression model, the following is obtained

13 $$-\Delta_{c}H^{o}\left(kJ\cdot mol^{-1}\right)=90\left(Estrada\right)-1.0789\left(Wiener\right)+0.2822\left(Gutman\right)-417.6882\left(kJ\cdot mol^{-1}\right)$$

A coefficient of determination of *R* ^2^ = 0.9760 is obtained. However, this fit is only marginally better than that achieved using the Estrada index alone and also presents a risk of collinearity among predictors. Therefore, it is more appropriate to use nonparametric ML models, which allow the integration of multiple descriptors with greater robustness against multicollinearity.

### Predictions with Topological Indices

Based on the previously described results, supervised machine learning algorithms were employed to improve the prediction of combustion enthalpy, using both the statistical properties of the graphs and the aforementioned topological indices as predictor variables. First, the data set was divided into two subsets: a training set and a test set. The training set comprised 70% of the total data, while the test set corresponded to the remaining 30%. The training subset was used to train and validate 19 regression models, employing 10-fold cross-validation as the evaluation technique and using *MAE*, *MAPE*, *RMSE*, *RMSLE*, and *R* ^2^ as evaluation metrics. The evaluation metrics for each model are presented in Table S2 in the Supporting Information section. Table 1 shows the validation metrics for the three best-performing models, where it can be observed that all three correspond to tree-based ensemble models (hyperparameters not listed were set to their default values according to the implementation in the Pycaret environment). However, the Random forest model is known for its low risk of overfitting, good scalability, and high interpretability.^90,135,136^ Although Light gradient boosting machine may outperform Random forest in terms of accuracy and speed when properly tuned, the Random forest model offers greater stability and reliability. For these reasons, it was selected as the baseline model.

**Table 1:** Mean and Standard Deviation of the Evaluation Metrics on the Validation Set from 10-Fold Cross-Validation for the Three Best-Performing Models^a^

Model	*R* ^2^	*MAE*	*MAPE*	*RMSE*	*RMSLE*
Random forest (estimators = 100, bootstrap = true)	0.9863 ± 0.0026	266.5924 ± 37.5274	0.1121 ± 0.0635	493.9055 ± 72.9987	0.1856 ± 0.0576
Extra trees regressor (estimators = 100, bootstrap = true)	0.9861 ± 0.0024	262.7963 ± 33.9388	0.1125 ± 0.0622	497.4291 ± 63.9609	0.1884 ± 0.0550
Light gradient boosting machine (estimators = 100, learning rate = 0.1, boosting = gbdt)	0.9860 ± 0.0021	299.6606 ± 27.5196	0.1222 ± 0.0681	498.6294 ± 59.0417	0.1857 ± 0.0592

a *MAE* and *RMSE* expressed in units of kJ·mol^–1^.

The initial analysis using the Random forest model identified the most important predictor variables, revealing that topological indices are the most significant for predicting the standard enthalpy of combustion (Gutman index = 0.4544, Estrada index = 0.4339, Wiener index = 0.0585, CC = 0.0344, Degree = 0.0126, EC = 0.0033, and BC = 0.0016). This finding is particularly relevant, as one of the main limitations of machine learning models is the difficulty in understanding how the model predicts the dependent variable from certain independent variables or predictors. Therefore, it is essential to employ XAI methods.^97^ To validate the identification of important variables obtained with the Random forest model, the SHAP method was also applied. This method is based on game theory, where, in the context of machine learning, the game is the predictive model, the players are the predictor variables, the payoff is the model prediction, and the objective is to fairly assign a contribution from each feature to the prediction. That is, SHAP assigns an additive contribution from each predictor variable to the model’s output, such that the sum of all contributions equals the predicted value. Therefore, the magnitude of each contribution reflects the importance of the corresponding predictor.^95^ The SHAP analysis strongly suggested that the Estrada, Gutman, and Wiener indices and Degree were the most important variables, based on both their ranking and the spread of SHAP values (see Figure 7).

**Figure 7:**
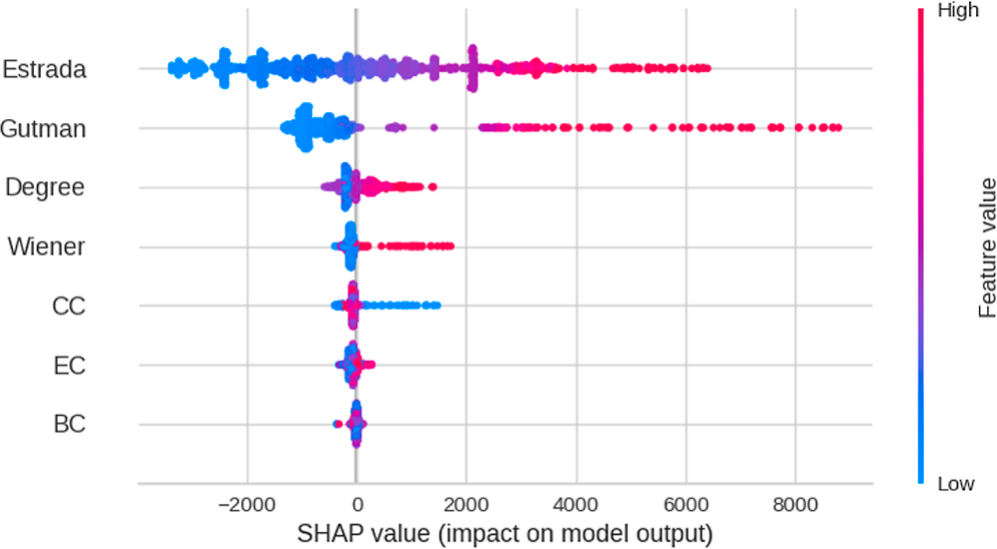
SHAP summary plot showing the contribution of topological descriptors to the prediction of standard combustion enthalpy. Each point represents a compound, and the color indicates the descriptor value (from low in blue to high in red). The Estrada index is observed to have the greatest impact on the model, followed by the Gutman index, highlighting the importance of global molecular connectivity in the prediction. Centrality-based descriptors (closeness, eigenvector, and betweenness) exhibit a lower influence on the model output.

High values of these topological indices tend to increase the model output, in agreement with the random forest results. Both the random forest model and the SHAP method identified the Estrada, Gutman, and Wiener indices as the most relevant predictors. As previously mentioned, the Estrada index is derived from the spectral properties of the molecular graph’s adjacency matrix, as it is calculated from the eigenvalues of this matrix, which are also used to compute the so-called graph energy.^137^ Therefore, this index reflects substructural centrality and the vibrational energy of the system. Additionally, it is closely related to the internal connectivity of atoms within a molecule and is influenced by the presence of highly interconnected substructures, such as aromatic rings or long branched chains, which directly affect the total energy content of the compound.^137^ Furthermore, it is linked to the three-dimensional structure of molecules through higher-order line graphs and thus to conformational stability, stored energy, and, consequently, to the amount of energy released during combustion. On the other hand, the Gutman index is calculated from the node degrees and the topological distances between them, thus incorporating information on both connectivity and structural dispersion. As a result, this topological index provides valuable information regarding bond density, structural compactness, and the total energy contained in the molecules. Therefore, more densely connected molecules tend to exhibit higher Gutman indices and, consequently, release more energy upon combustion. As for the Wiener index, it incorporates only the sum of the shortest path lengths between all pairs of atoms in each molecule. It provides general information about molecular compactness but does not capture electronic or energetic aspects. Both the Estrada and Gutman indices indirectly encapsulate critical information about bond lengths and energies, degree of branching, electronic density, steric and volumetric distribution, and, therefore, reactivity toward oxygen. In other words, they capture relevant topological, energetic, and structural information, which explains why the Random forest model and SHAP method assign the greatest contribution to these variables in predicting combustion enthalpy.

Once the most important variables were identified, these features were used as predictor variables, and a random grid search with 5-fold cross-validation was performed to tune the hyperparameters of a Random forest model. The results of the hyperparameter tuning process, learning and validation curves, residual plots, and feature importance analysis are available in the GitHub repository for this document (https://github.com/jarzolads/PredictionEnthalpy). Figure 8 shows the parity plots for the training and test sets, and Table 2 presents the evaluation metrics using the entire training subset (70%) and the test subset (30%). In both cases, good predictions of standard combustion enthalpy were obtained, indicating that topological indices are strong predictors of this thermochemical property.

**Figure 8:**
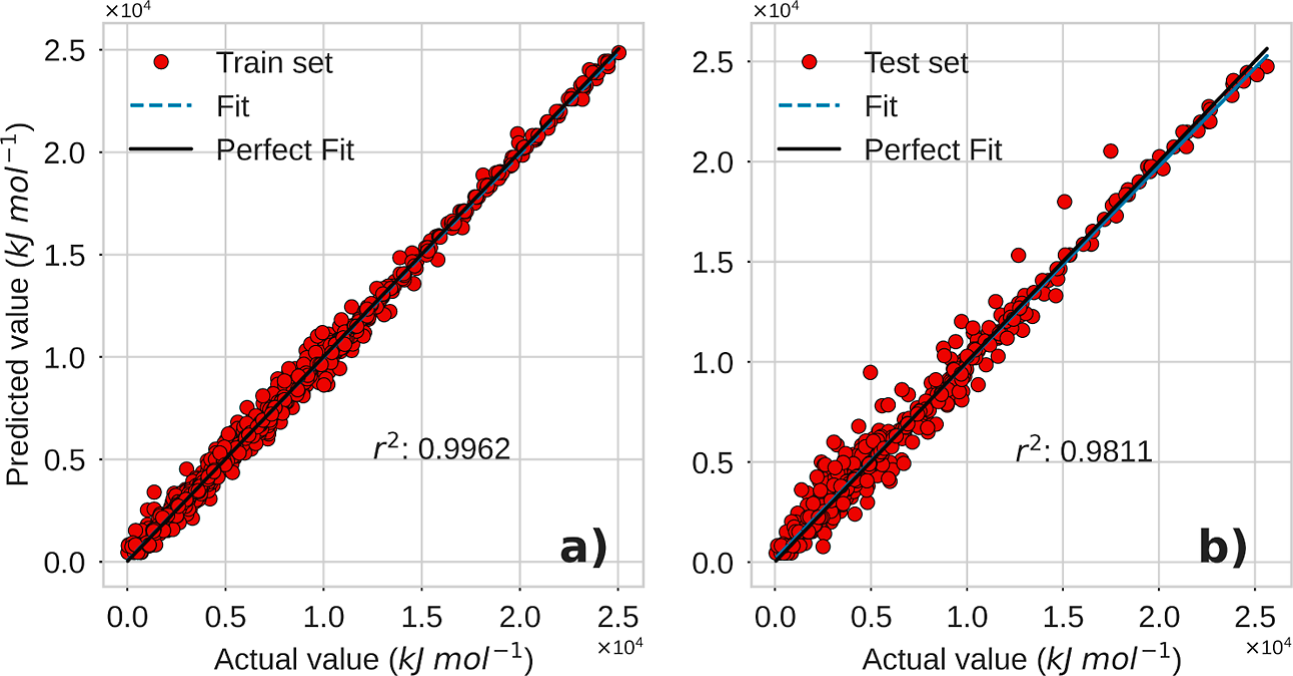
Parity plots of the prediction. (a) Training set. The *r* ^2^ = 0.9962 value corresponds to the fit of predicted vs actual values, and (b) test set. In both cases, a good linear relationship is observed between the actual combustion enthalpy values and the values obtained from the random forest model prediction.

**Table 2:** Evaluation Metrics Obtained With the Random Forest (Estimators = 100, Bootstrap = True) Model Using the Topological Indices of Graphs Constructed from Organic Molecules as Predictors^a^

Set	*R* ^2^	*MAE*	*MAPE*	*RMSE*	*RMSLE*
Train	0.9962	142.9970	0.0661	261.1587	0.1462
Test	0.9810	287.5988	0.1048	551.9050	0.1933

a *MAE* and *RMSE* expressed in units of kJ·mol^–1^.

### QSPR Models for Families of Compounds

Numerous research studies have investigated QSPR models employing topological indices based on vertex degree and interatomic distances as predictors of physicochemical properties.^53,60,72^ However, only a limited number of studies have explored the use of spectral indices, such as the Estrada index. Consequently, QSPR models were developed for selected families of compounds within the complete data set.

QSPR models for aromatic compounds (111 compounds) were constructed using the Extra trees (ET) algorithm. Additionally, acyclic alkanes (502), cycloalkanes (116), acyclic alkenes (175), cycloalkenes (67), and alkynes (82) were modeled using Huber regression. Unlike ordinary least-squares regression, Huber regression minimizes a loss function that is quadratic for small residuals and linear for large residuals.^138^ This behavior allows the model to maintain efficiency when the errors are small while simultaneously reducing the influence of outliers that may bias the fit. In practice, this behavior is controlled by the ϵ hyperparameter, while *L*_2_ regularization is tuned through the α parameter. These properties make Huber regression both accurate and robust in the presence of noisy data or outliers.

Finally, linear regression models were used to construct QSPR models for the prediction of standard combustion enthalpy for amines (21 acyclic compounds: 15 primary, 4 secondary, and 2 tertiary) and for acyclic carboxylic acids. For the latter, 10 compounds containing a single carboxyl group and 16 compounds with two carboxyl groups in their structure were used. It is worth noting that previous studies have addressed the investigation of certain thermochemical properties of these carboxylic acids.^38,39^

For aromatic compounds, acyclic alkanes, cycloalkanes, acyclic alkenes, cycloalkenes, and alkynes, model evaluation was performed using nested cross-validation with five outer folds and three inner folds. In all cases, the performance metrics included *R* ^2^, *MAE*, *MAPE*, *RMSE*, and *RMSLE*. For those models evaluated using nested cross-validation, the mean and standard deviation of each metric are reported. The results are presented in Table 3, and the corresponding parity plots are shown in Figure 9.

**Table 3:** Evaluation Metrics for Various Models Applied to Different Organic Compound Families^a^

Family	Model	*R* ^2^	*MAE*	*MAPE*	*RMSE*	*RMSLE*
Aromatics	ET (estimators = 100, bootstrap = true)	0.9998 ± 0.000166	21.109760 ± 17.329510	0.0015 ± 0.000896	51.2618 ± 49.3509	0.003198 ± 0.0020
Acyclic alkanes	Huber (α = 0.0001, ϵ = 1.8)	0.9999 ± 0.000006	5.7638 ± 0.3214	0.00096 ± 0.00021	8.6844 ± 0.8478	0.0020 ± 0.0013
Cycloalkanes	Huber (α = 0.7, ϵ = 1.2)	0.9999 ± 0.000004	17.8765 ± 3.4568	0.0034 ± 0.000870	26.5120 ± 2.2231	0.0062 ± 0.0011
Acyclic alkenes	Huber (α = 0.01, ϵ = 1)	0.9999 ± 1.8497 × 10^–15^	1.7058 × 10^–4^ ± 4.8641 × 10^–5^	2.6724 × 10^–8^ ± 3.0067 × 10^–9^	2.7109 × 10^–4^ ± 1.0251 × 10^–4^	2.8497 × 10^–7^ ± 3.0336 × 10^–8^
Cycloalkenes	Huber (α = 1 × 10^–7^, ϵ = 1.1)	0.9999 ± 0.0000	5.4723 × 10^–3^ ± 1.0076 × 10^–2^	8.6131 × 10^–7^ ± 1.5327 × 10^–6^	6.7603 × 10^–3^ ± 1.2536 × 10^–2^	1.0988 × 10^–6^ ± 1.5927 × 10^–6^
Alkynes	Huber (α = 1 × 10^–7^, ϵ = 1.1)	0.9999 ± 0.000002	9.7645 ± 0.7086	0.0017 ± 0.0005	12.5305 ± 1.3296	0.0026 ± 0.0013
Amines	Linear regression	0.9997	39.8430	0.0041	46.8209	0.0049
Carboxylic acids	Linear regression (one and two groups)	one group: 0.9999, two groups: 0.9999	one group: 9.6278, two groups: 14.8646	one group: 0.0011, two groups: 0.0162	one group: 13.0146, two groups: 20.1506	one group: 0.0017, two groups: 0.0537

a *MAE* and *RMSE* expressed in units of kJ·mol^–1^.

**Figure 9:**
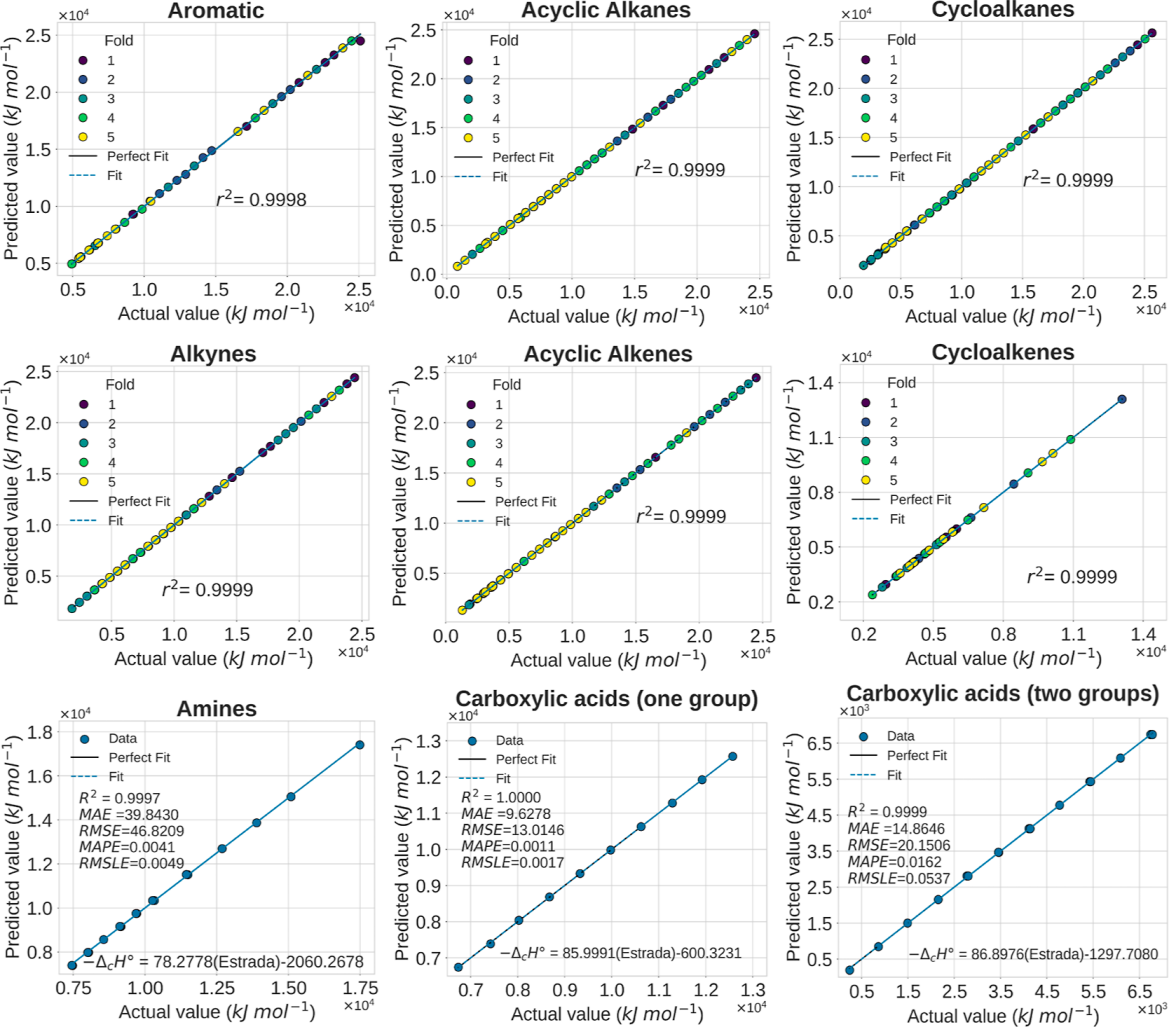
Comparison between the actual and predicted values of the standard enthalpy of combustion for different families of organic compounds using regression models based on the Estrada index. The six upper plots show the results of nested cross-validation across the five outer folds for aromatic compounds, acyclic alkanes, cycloalkanes, alkynes, acyclic alkenes, and cycloalkenes. The three lower plots present individual fits for amines and carboxylic acids (considering the presence of one or two carboxyl groups), including the *R* ^2^, *MAE*, *RMSE*, *MAPE*, and *RMSLE* metrics, as well as the corresponding linear regression equations. The results highlight the accuracy of the models and the relevance of the Estrada index as a topological descriptor in QSPR studies.

As previously mentioned, earlier studies have addressed the prediction of thermochemical properties of carboxylic acids, such as the standard enthalpy of formation in the crystalline and gaseous phases, using tailored group contribution methods based on machine learning. These studies compared their predictions with both experimental values and estimates obtained using the classical Benson group contribution method, reporting satisfactory results.^38,39^ In this context, a comparison was carried out between the standard combustion enthalpy predicted using the Estrada index employing the corresponding regression equation for compounds with one or two carboxyl groups and the estimates obtained via the Benson method as well as the experimental data (see Table 4). As shown in the table, the results are comparable and, in some cases, even superior.

**Table 4:** Comparison of the Experimental Combustion Enthalpy with the Values Estimated Using the Benson Group Contribution Method and Those Predicted by the QSPR Model Based on the Estrada Index, Including the Relative Errors

Compound	$\Delta_{c}H_{\exp}^{o}\left(kJ\cdot mol^{-1}\right)$	Δ_c_ *H*_Benson_^o^ (kJ·mol^–1^)	Δ_Benson_ (kJ·mol^–1^)	Δ_c_ *H_pred_^o^* (kJ·mol^–1^)	Δ_pred_ (kJ·mol^–1^)
Undecanoic acid	6736.5	6727.07	–9.43	6743.4694	6.97
Dodecanoic acid	7423.7	7377.00	–46.60	7391.1249	–32.58
Tridecanoic acid	8024.2	8026.93	2.73	8038.7803	14.58
Tetradecanoic acid	8676.7	8676.86	0.16	8686.4358	9.74
Pentadecanoic acid	9327.7	9326.79	–0.91	9334.0912	6.39
Succinic acid	1491.0	1504.23	13.23	1503.8081	12.81
Sebacic acid	5425.0	5403.81	–21.19	5430.3442	5.34
Pimelic acid	3460.2	3454.02	–6.18	3467.0760	6.88
Trimethylsuccinic acid	3450.75	3460.98	10.23	3467.6408	16.89
Triethylsuccinic acid	5441.3	5421.81	–19.49	5430.9194	–10.38

Based on the above, it is possible to state that topological indices offer significant advantages over traditional group contribution methods, particularly in terms of automation, applicability to structurally diverse compounds (provided that a suitable data set is available), and ease of integration with ML models. These topological indices, derived from the molecular structure, enable the capture of global connectivity features without the need for manual assignment of functional groups, an important advantage given that, in some cases, group contribution parameters may not be available. However, their physicochemical interpretation can be less straightforward, highlighting the need for XAI methods.

In contrast, the Benson method is based on well-established empirical principles, offering high interpretability and strong physicochemical grounding. Nevertheless, its applicability may be limited when dealing with compounds containing unparameterized functional groups or novel structures.^25^

Based on this comparison, it is worth emphasizing that the strategy proposed in this research using topological indices to build QSPR models for the standard enthalpy of combustion provides a flexible and scalable alternative for predicting thermochemical properties such as combustion enthalpy, demonstrating performance comparable to that of traditional approaches.

### Predictions with Molecular Descriptors

To validate the results obtained using topological indices as predictive variables, 210 molecular descriptors of each of the 3477 compounds were employed as predictors of combustion enthalpy. These descriptors were generated using the RDKit Python library. Multiple supervised learning models were trained following the same strategy used for the models based on topological indices, that is, using the same data partitioning scheme, evaluation method, and performance metrics (the results are presented in Table S3 in the Supporting Information section).

The results indicate that Ridge regression (α = 1) is a suitable model for predicting standard combustion enthalpy (validation set metrics: *R* ^2^ = 0.9979 ± 0.0009, *MAE* = 88.1622 ± 5.4378, *MAPE* = 0.0349 ± 0.0140, *RMSE* = 187.5298 ± 26.0710, *RMSLE* = 0.1119 ± 0.0504). However, to mitigate multicollinearity effects and to enable a direct comparison with the results obtained using topological indices as predictor variables, the Random forest (validation set metrics: *R* ^2^ = 0.9936 ± 0.0030, *MAE* = 143.6763 ± 12.9692 kJ/mol, *MAPE* = 0.0661 ± 0.0506, *RMSE* = 325.4404 ± 52.3858 kJ/mol, *RMSLE* = 0.1261 ± 0.0640) model was employed once again. Similarly, it was found that the most important predictor variables correspond to the Chi1n (0.6072) and Chi0n (0.2525) indices, which was further confirmed through SHAP analysis.

After selecting the Random forest model, the hyperparameters of the models were tuned using a random grid search with 5-fold cross-validation. The results of the hyperparameter tuning process, learning and validation curves, residual plots, and feature importance analysis can be found in the code available in the GitHub repository for this article (https://github.com/jarzolads/PredictionEnthalpy). Parity plots for both the training and test sets are shown in Figure 10. The evaluation metrics are presented in Table 5.

**Figure 10:**
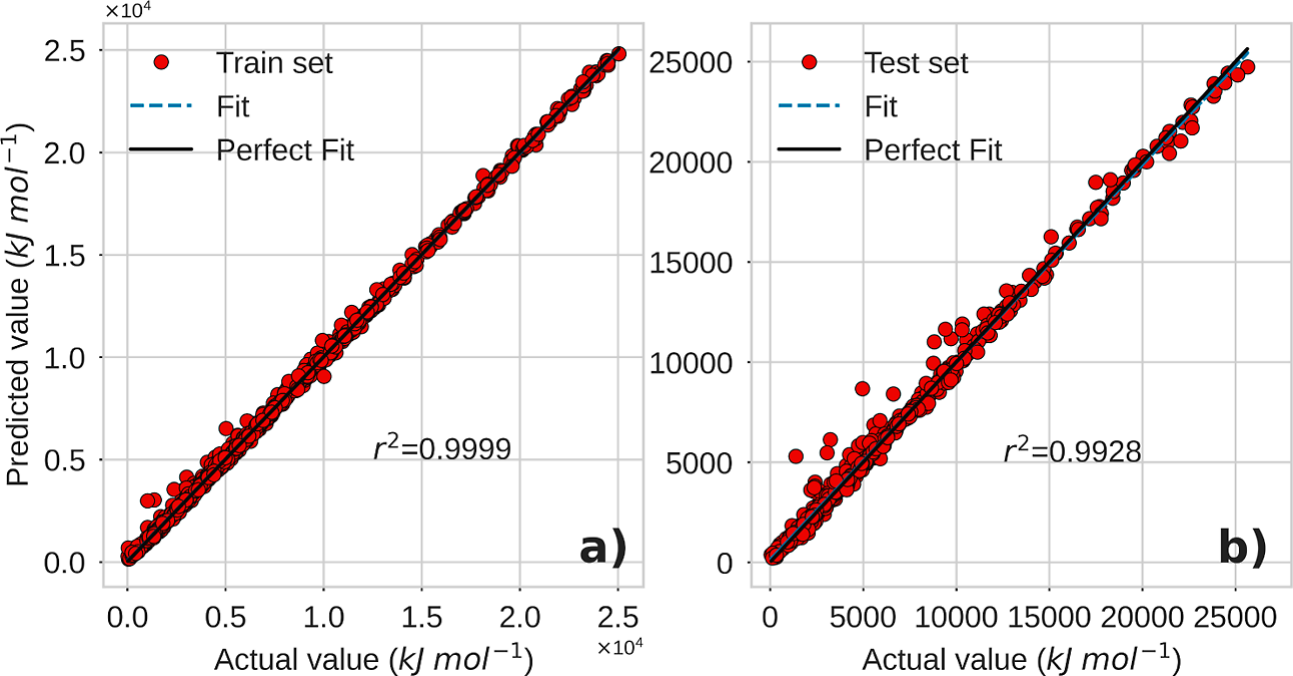
Prediction parity plots. (a) Training set. The *r* ^2^ = 0.9999 value corresponds to the fit of predicted vs actual values, and (b) test set (*r* ^2^ = 0.9928).

**Table 5:** Evaluation Metrics Obtained with the Random Forest Model (Estimators = 100, Bootstrap = True) Using RDKit Descriptors of Organic Molecules as Predictors^a^

Set	*R* ^2^	*MAE*	*MAPE*	*RMSE*	*RMSLE*
Train	0.9991	51.5854	0.0243	122.1867	0.0855
Test	0.9927	142.2272	0.0484	342.0464	0.1172

a *MAE* and *RMSE* expressed in units of kJ·mol^–1^.

By comparing the results shown in Tables 2 and 5, it is observed that the evaluation metrics for the test set are slightly better when using the 210 RDKit molecular descriptors as predictors. However, using only three topological indices derived from the graphs of organic molecules yields similar results, indicating that the topological indices encapsulate information contained in the RDKit molecular descriptors.

The top 30 most significant correlations between molecular descriptors and topological indices are listed in Figure 11. As shown, the Estrada index is strongly correlated with the connectivity indices Chi0n, Chi1n, and Chi2n, as both quantify atom connectivity in terms of single bonds and are dependent on the number and distribution of bonds.^139–142^ This indicates that highly interconnected organic molecules tend to have higher values for this topological index due to greater substructural centrality. Additionally, the SlogP–VSA and SMR–VSA descriptors are related to the accessible surface area and the potential solubility of the molecule,^143^ and their high correlation with the Estrada index comes from the fact that molecules tend to have greater internal connectivity, which is reflected by this index.

**Figure 11:**
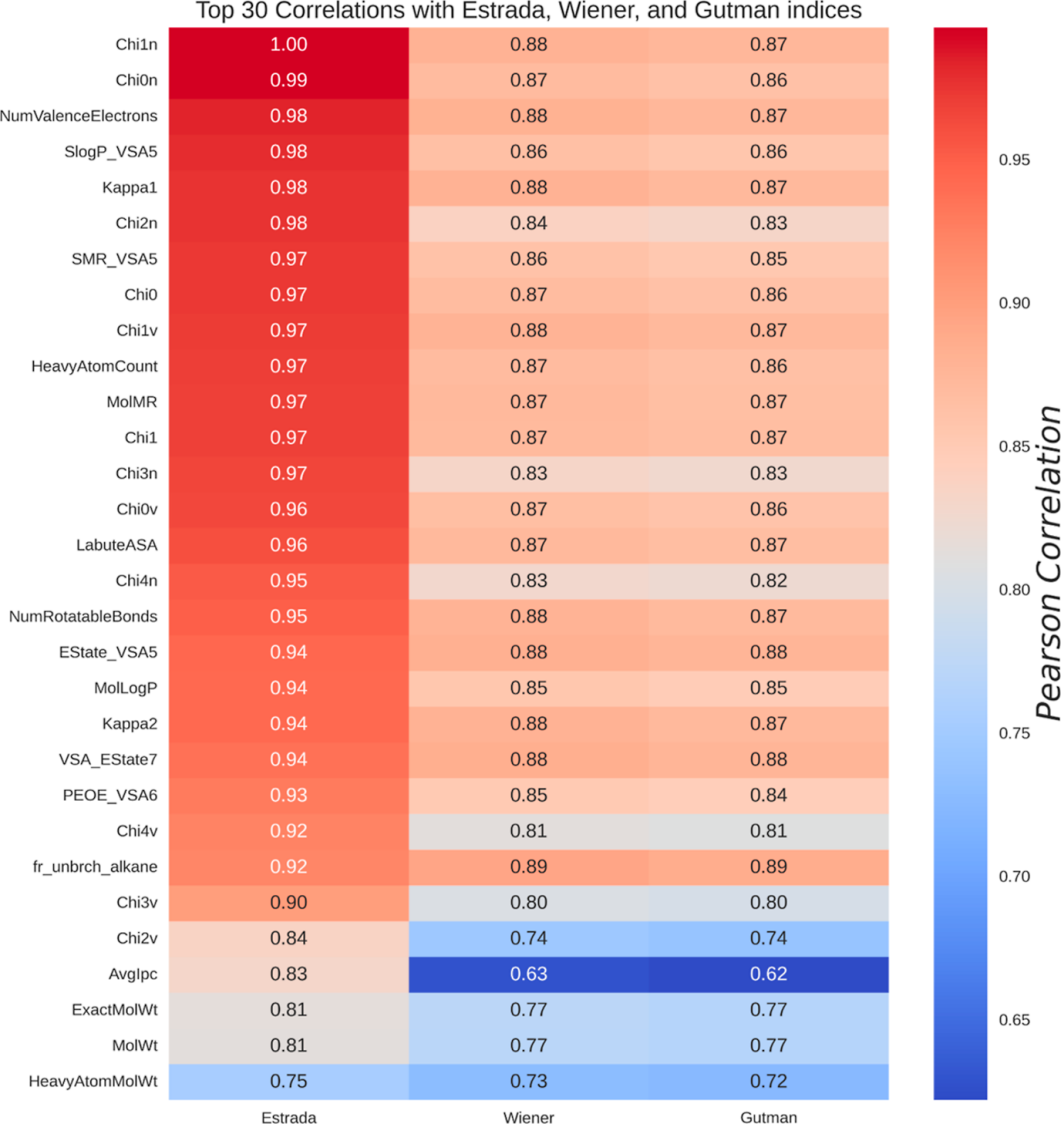
Top 30 strongest correlations between RDKit molecular descriptors and topological indices. The figure shows a strong positive correlation between various molecular descriptors and the Estrada index. In particular, the descriptors Chi1n, Chi0n, and the number of valence electrons exhibit Pearson correlations greater than 0.95 with the Estrada index. These results support the structural relevance of topological indices in the analysis of molecular properties.

This relationship is significant, as these molecular descriptors are important in drug design to study biological activity, suggesting that the Estrada index could also be used in drug design.^137^ There is also a high correlation with the total number of valence electrons (NumValenceElectrons), a molecular descriptor that is indirectly related to a molecule’s reactivity and stability, supporting the idea that the Estrada index can serve not only to study molecular properties related to connectivity but also molecular reactivity, especially in structures where valence electrons are distributed across a complex network of bonds.

Regarding to the Kappa descriptors, these measure the degree of branching in a molecule,^139,144^ which implies high connectivity and, thus, a high Estrada index. Additionally, these molecular descriptors are associated with the combustion enthalpy of an organic compound due to various structural and electronic factors affecting the energy released during combustion.

For instance, the connectivity indices Chi0, Chi1n, and Chi2n measure connectivity within a molecule, considering the amount of energy stored in the molecular structure, as the energy released in combustion involves breaking and forming chemical bonds. Chi indices are also associated with electronic density,^139^ meaning that molecules with higher electronic density are often more reactive, leading to greater energy release in combustion processes. This indicates that high Chi index values correspond to higher Estrada index values and, therefore, higher combustion enthalpy values.

Similarly, combustion enthalpy is directly related to the number of valence electrons, as these participate in chemical reactions.^27,133^ A molecule with more valence electrons can form or break more bonds during combustion. Thus, more valence electrons suggest a potential for stronger bonds with oxygen during combustion,^143^ leading to increased combustion enthalpy. An increase in the number of valence electrons implies an increase in the Estrada index and, consequently, an increase in combustion enthalpy.

Moreover, molecules with a more accessible surface area or larger volume (quantified by the SlogP–VSA and SMR–VSA descriptors) can be more reactive, since having more accessible surfaces implies a potential increase in reactivity with oxygen in combustion. The greater the magnitude of this molecular descriptor, the higher the value of the Estrada index and combustion enthalpy. Finally, more branched molecules (quantified by the Kappa indices) often have secondary and tertiary bonds that are possibly less stable and more susceptible to breaking during combustion, meaning that molecules with high Kappa values release more energy by breaking these less stable bonds, leading to a higher combustion enthalpy and a higher Estrada index.

All of this reinforces that it is possible to predict combustion enthalpy for organic molecules with high accuracy using topological indices derived from the graphs representing these molecules.

Figure 11 also shows a strong positive correlation between the Chi1n and Chi0n indices with the Wiener index. This is because Chi indices reflect how atoms are connected within a molecule, and the Wiener index, being a cumulative measure of distances between pairs of nodes (atoms within a molecule), will increase for structures with high connectivity and, consequently, high Chi index values. Additionally, molecules with high connectivity or extensive branching tend to have shorter paths between many pairs of nodes, which increases the total sum of distances (Wiener index).

These molecules will also have high Kappa index values, which directly influence the Wiener index. Molecules with a high number of valence electrons will have elevated volumetric properties such as SlogP–VSA and SMR–VSA, which also implies more bonds and a higher sum of distances between pairs of nodes (atoms) that lead to an increase in the Wiener index. Lastly, the Gutman index, derived from the weighted sum of distances between nodes, also depends on the connectivity of the graph that represents each molecule and correlates with the previously described molecular descriptors.

### Clustering Analysis with Topological Indices

Based on the results obtained so far, it can be stated that topological indices encompass information about molecular descriptors, making it plausible to consider a topological space that groups organic compounds according to the magnitude of the Estrada, Wiener, and Gutman indices. This approach aims to explore potential latent patterns within this space. A clustering analysis not only facilitates the structural interpretation of thermochemical behavior but also supports the design of more robust predictive models by considering groups of compounds with similar thermochemical and topological properties. This makes it reasonable to employ unsupervised learning techniques to identify potential clusters. The optimal number of clusters was identified with the elbow method^145^ for the K-Means algorithm (see Figure 12). The following hyperparameters were used: K-Means++ initialization method, one initialization, Lloyd’s algorithm for determining distances, and varying the number of clusters. In this graph, the closer the sum of squared distances to the nearest cluster center is to zero, the better the clustering.

**Figure 12:**
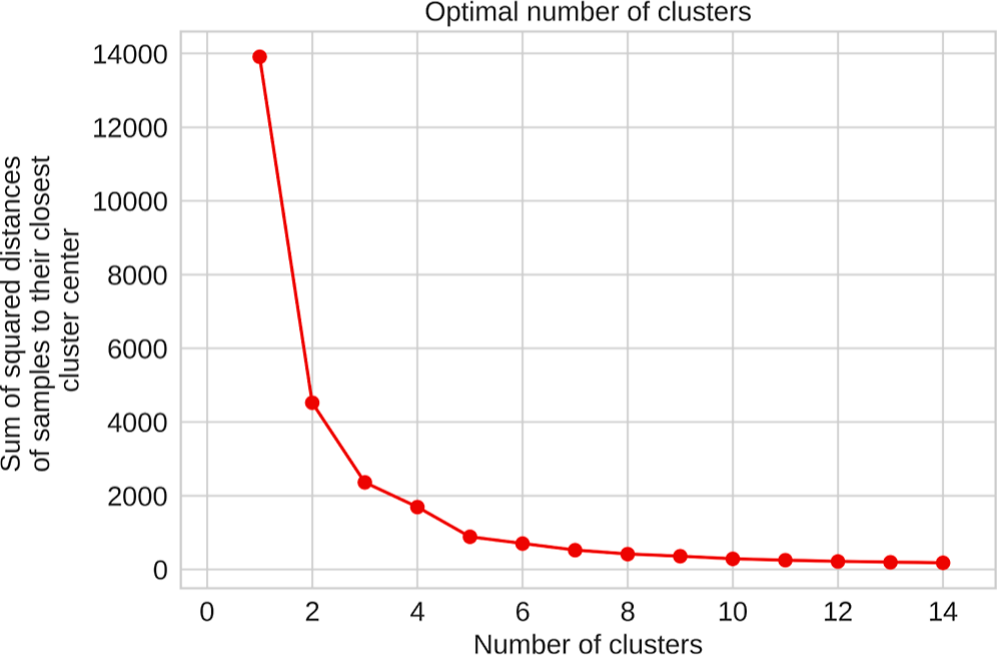
Elbow method plot used to determine the optimal number of clusters in a clustering analysis with the K-means algorithm. The vertical axis represents the sum of squared distances between each sample and the centroid of its nearest cluster. As the number of clusters increases, this sum decreases.

The graph shows that using more than ten clusters reduces the sum of squared distances to zero. However, at this point, two questions arise: How suitable is K-Means for clustering? And how appropriate is it to choose more than ten clusters? To answer the first question, the K-Means algorithm was compared with the DBSCAN algorithm (hyperparameters: maximum distance between two samples of 0.1, 20 samples, and Euclidean metric, with an average silhouette coefficient of 0.081 and a Davies-Bouldin score of 1.0367) and the HDBSCAN algorithm (hyperparameters: minimum number of samples in a cluster of 50, with an average silhouette coefficient of −0.09 and a Davies-Bouldin score of 2.0875). The K-Means algorithm showed better evaluation metrics than the others (average silhouette coefficient of 0.5033 and a Davies-Bouldin score of 0.5606).

To address the second question. The K-Means algorithm was defined using the hyperparameters: K-Means++ initialization method, one initialization, and Lloyd’s algorithm; the number of clusters was varied, and the silhouette coefficient was obtained to evaluate clustering quality. It was found that using seven clusters resulted in an average silhouette coefficient of 0.5033 and a Davies-Bouldin score of 0.5606. It is important to note that the closer the silhouette coefficient is to 1 and the Davies-Bouldin score is to zero, the better the clustering. Figure 13 shows the silhouette analysis for the clustering model with seven clusters, which yields an average silhouette coefficient slightly above 0.5, indicating a moderately well-defined cluster structure. Some clusters exhibit a broad distribution of silhouette values, with most coefficients exceeding 0.4, suggesting good internal cohesion and separation from other groups. This observation is further supported by the Davies–Bouldin coefficient.

**Figure 13:**
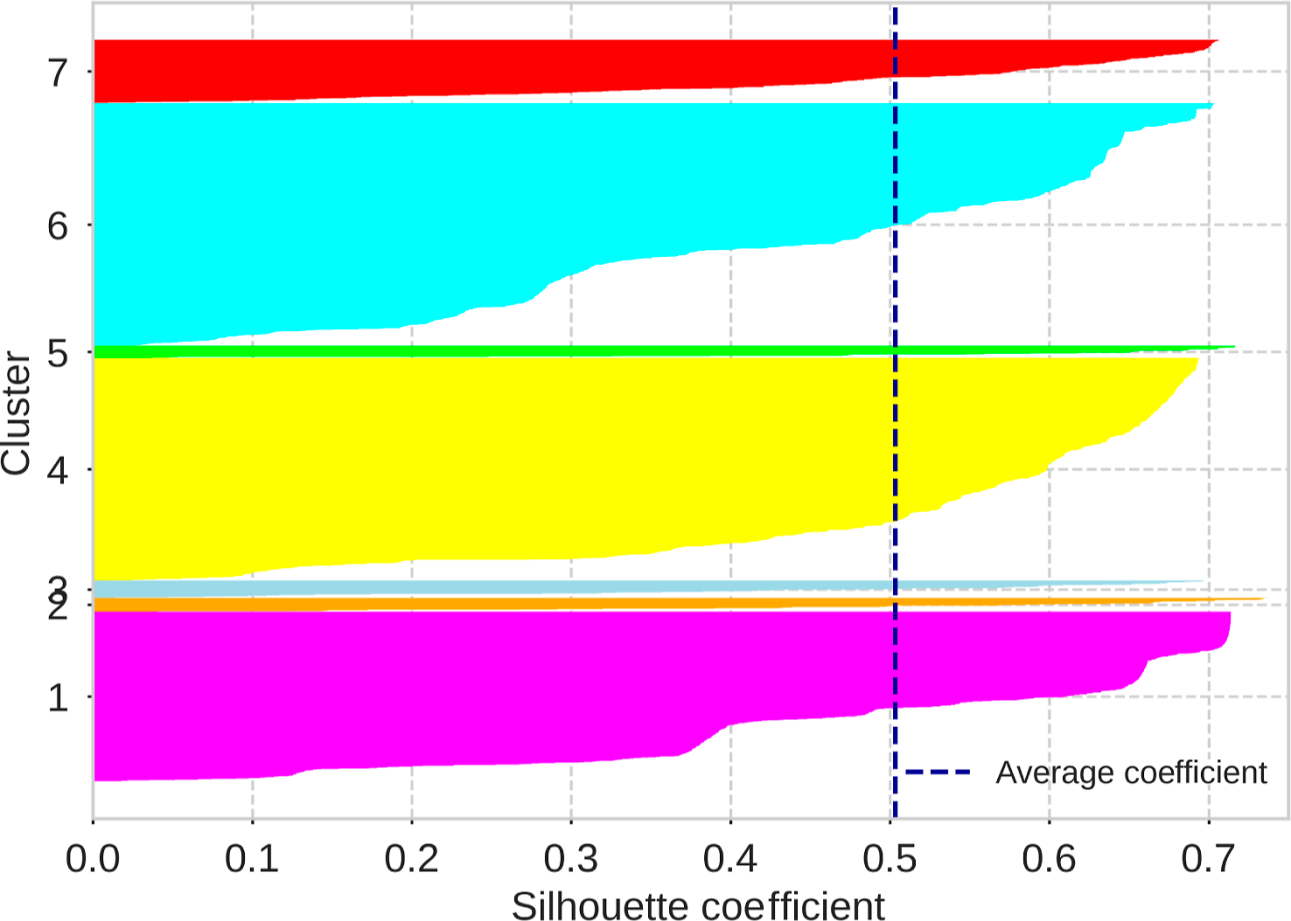
Silhouette coefficient plot for the clustering into 7 clusters. Each bar represents the individual silhouette coefficient of a sample within its assigned cluster. The colors visually distinguish the different groups (clusters 1 to 7). The horizontal axis indicates the silhouette coefficient value, which measures how similar a sample is to its own cluster compared to other clusters. Values close to 1 indicate a well-assigned sample, while values near 0 suggest ambiguity in the clustering. The dashed line represents the average silhouette coefficient, which in this case is slightly above 0.5, indicating a reasonably well-defined cluster structure.

Figure 14 shows the clustering of organic compounds in the topological space formed by the Estrada, Wiener, and Gutman indices, while Figure 15 presents the standard combustion enthalpy as a function of the Estrada, Wiener, and Gutman indices for each cluster. As observed in Figure 15a,d, as the Estrada index increases, so does the standard combustion enthalpy in each cluster. This, as mentioned earlier, is because this index measures the structural properties of a graph, providing information about connectivity and bond distribution. In other words, molecules with greater connectivity and more bonds exhibit higher values of standard combustion enthalpy.

**Figure 14:**
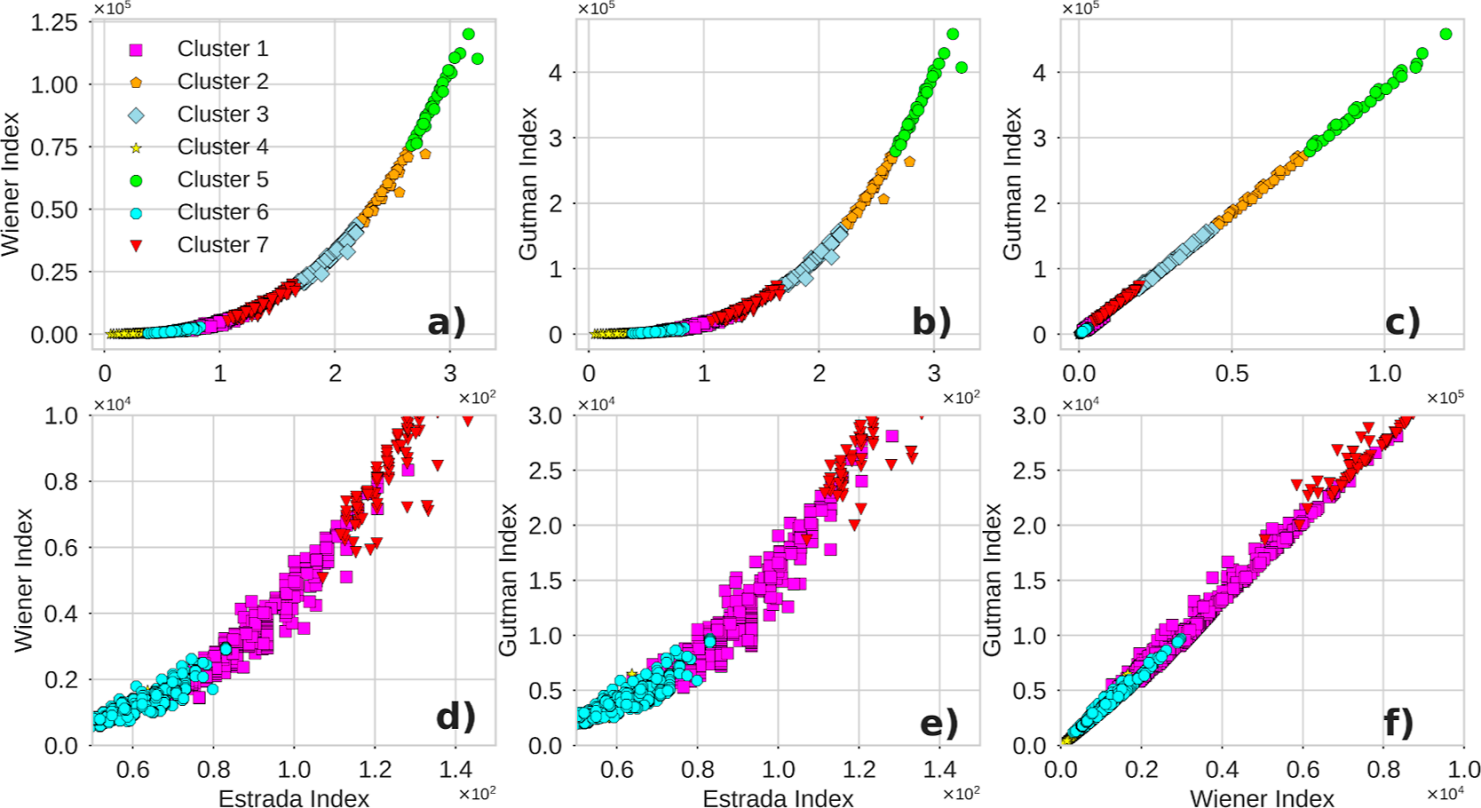
Topological space formed by the Estrada, Wiener, and Gutman topological indices. Figure (a) shows the space formed by the Wiener and Estrada indices; Figure (d) provides a zoomed-in view of the region containing clusters 6, 1, and 7. In Figure (b), the space formed by the Gutman and Estrada indices is depicted, while Figure (e) is another zoomed-in view of the region with clusters 6, 1, and 7. Finally, Figure (c) represents the space formed by the Wiener and Gutman indices, and Figure (f) again focuses on the same cluster region.

**Figure 15:**
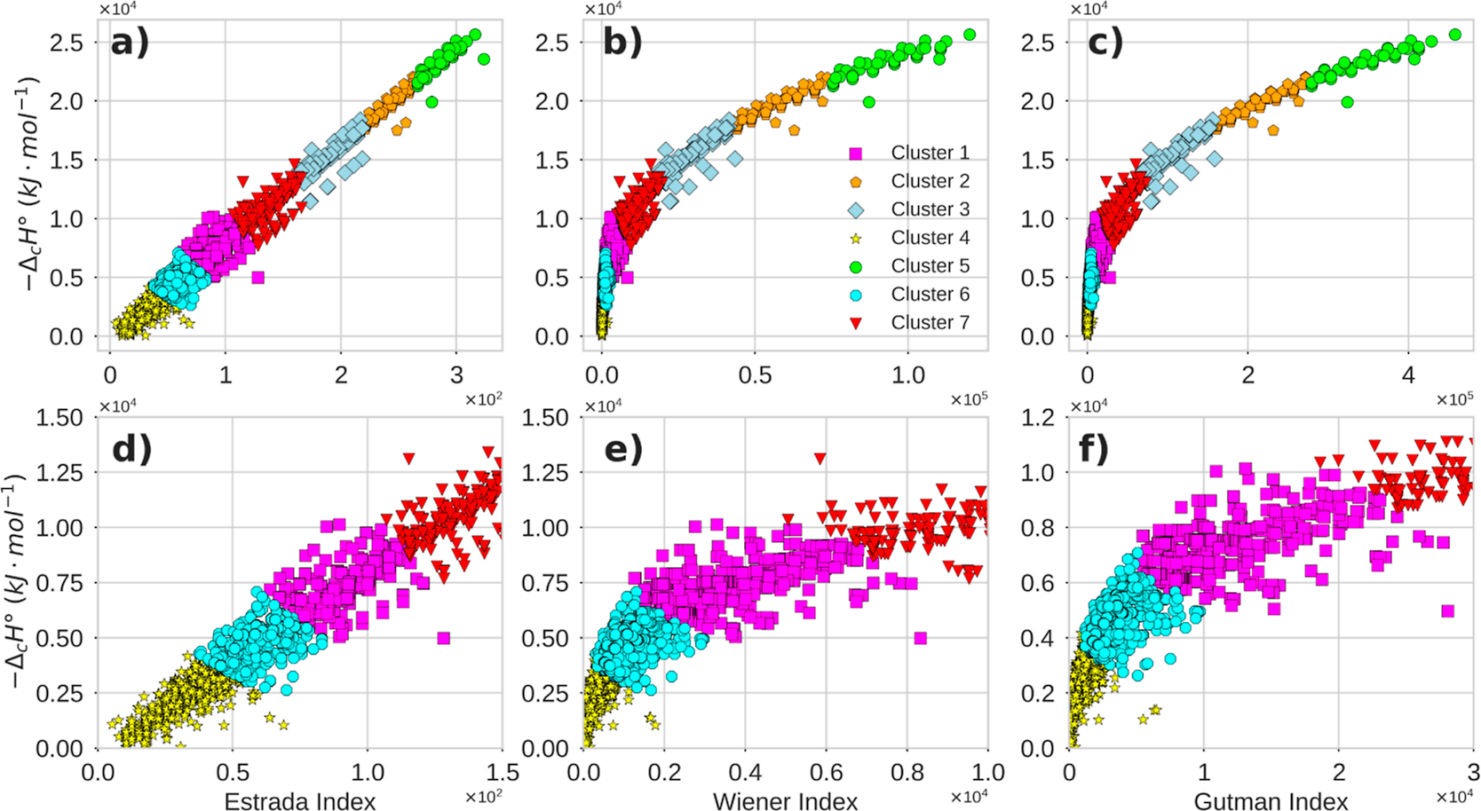
Standard combustion enthalpy as a function of the Estrada, Wiener, and Gutman indices for each cluster. Figure (a) shows that as the magnitude of the Estrada index increases, the combustion enthalpy value also increases. Figure (d) provides a zoomed-in view of the region containing clusters 4, 6, 1, and 7, highlighting the separation between clusters. A similar trend is observed in Figure (b), where an increase in the Wiener index corresponds to an increase in standard combustion enthalpy, while Figure (e) provides a closer look at the region containing clusters 4, 6, and 7. Figures (c,f) depict the behavior of combustion enthalpy as a function of the Gutman index, showing that higher values of this index are associated with higher combustion enthalpy.

A similar trend is observed for combustion enthalpy as a function of the Wiener index (Figure 15b,e) and the Gutman index (Figure 15c,f). Table S4 provides a descriptive statistical analysis and the 95% and 99% confidence intervals for each variable in each cluster (standard combustion enthalpy in kJ·mol^–1^).

It is clear at this point that topological indices allow clustering organic compounds together and that they strongly correlate with molecular descriptors (see Figure 11). This raises the question of what common characteristics these organic compounds share in terms of molecular descriptors. The results of the descriptive analysis and the 95% and 99% confidence intervals for the top five correlations from Figure 11 are presented in Table S5.

The magnitude of these descriptors follows a similar pattern to that of the topological indices for each cluster (see Table S4). For instance, higher magnitudes of the topological indices correspond to higher magnitudes of the descriptors Chi10, Chi0n, NumValenceElectrons, SlogP–VSA5, and Kappa1, and consequently, higher values of standard combustion enthalpy. In this case, Cluster 5 exhibits the highest magnitudes for these molecular descriptors.

The Chi1n and Chi0n indices are based on the connectivity of the molecule from an electronic perspective and are used to characterize the molecular structure by specifying the electronic identity of each atom.^139–141^ These indices also include information regarding the number of valence electrons (NumValenceElectrons).^113,139,144^ On the other hand, the Kappa1 index is used to characterize the shape of a molecule, meaning that different structural isomers have different values for this index, as it measures the “cyclicity” of a molecule.^133,139,141^ Another example of this type of index is Kappa2, which also shows a high correlation with the Estrada (*r* = 0.94), Wiener (*r* = 0.88), and Gutman (*r* = 0.87) indices (see Figure 11) and contains information about the spatial density of atoms in the molecule. Kappa indices are related to descriptors such as SlogP–VSA and SMR–VSA, which explains why these molecular descriptors—along with the Estrada, Wiener, and Gutman indices—can be used as predictors of the standard enthalpy of combustion.

Given that many molecular descriptors encode electronic, geometric, and functional information about compounds, their inclusion in the analysis enables a more comprehensive classification of chemical space. This not only enhances the ability to interpret clusters in terms of structural and functional similarity but also allows for the identification of regions within the space defined by topological indices where predictive models may behave differently. Consequently, this strategy can support the development of more specialized QSPR models (even for other properties such as logP, toxicity, etc.), improve model applicability assessment, and help detect outlier compounds or those outside the modeled chemical domain. Moreover, it facilitates inverse molecular design; for instance, if specific clusters are associated with high combustion enthalpies (such as cluster 5) or other desirable properties, it becomes possible to search for or design compounds with similar characteristics that fall within that chemical region.

For example, a general analysis based on the average values of the molecular descriptors most correlated with the topological indices (Chi1n, Chi0n, SlogP–VSA5, NumValenceElectrons, and Kappa1) revealed distinctive structural profiles for each cluster, which allow inference of potential industrial applications. Cluster 1, with intermediate values of Chi1n (5.45) and Chi0n (9.18), reflects moderate molecular graph connectivity. Its average NumValenceElectrons (≈74) indicates medium electronic density, while the average SlogP–VSA5 value (57.22) suggests the presence of nonpolar regions relevant to solubility in organic media. Furthermore, its moderate Kappa1 value (11.73) implies a moderately branched molecular shape. These characteristics suggest potential uses as pharmaceutical intermediates, functional solvents, or fuel additives. Representative compounds include methyldecylamine, ethylnonylamine, and 1,1-difluorodecane.

Cluster 2 exhibits high values for all descriptors (Chi1n = 15.89, Chi0n = 23.17, NumValenceElectrons ≈ 196, SlogP–VSA5 ≈ 196.95, Kappa1 = 32.18), indicating the presence of large, densely connected molecules, although structurally less complex than those in cluster 5. These features suggest applications in controlled-release systems or as high-molecular-weight bioactive compounds. Examples include 1-tritriacontyne, 1-tritriacontene, and 1-fluorodotriacontane.

Cluster 3 (Chi1n = 12.50, Chi0n = 18.45, NumValenceElectrons ≈ 157, SlogP–VSA5 = 152.22, Kappa1 = 25.60) contains large, branched molecules that are suitable for optoelectronic materials, rheological modifiers, or functional inks. Representative compounds include 1-hexacosene, 1-fluoropentacosane, and 1-chloropentacosane.

Cluster 4 shows the lowest values for molecular descriptors (Chi1n = 2.07, Chi0n = 4.05, NumValenceElectrons ≈ 39, SlogP–VSA5 = 12.72, Kappa1 = 6.11), indicating small molecules with low connectivity and simple geometries. Examples include 4-pentenoic acid, 1,4-butanediol, and 1,4-difluorobutane. Based on these features, compounds in this cluster may serve as synthesis intermediates, platforms for chemical functionalization, or organic solvents.

Cluster 5 displays the highest values across all descriptors (Chi1n = 18.80, Chi0n = 27.30, NumValenceElectrons ≈ 232, SlogP–VSA5 = 234.69, Kappa1 = 38.12), suggesting that these compounds may be useful as energetic materials, industrial adhesives, or functional coatings. Examples include 1-nonatriacontyne, 1-chloroheptatriacontane, and 1-fluoroheptatriacontane.

Cluster 6 exhibits low-to-intermediate descriptor values (Chi1n = 3.61, Chi0n = 6.4, NumValenceElectrons ≈ 53, SlogP–VSA5 = 31.48, Kappa1 = 7.98), and includes functionalized alkyl aromatics. These structures are suitable for applications such as viscosity modifiers, low-polarity solvents, or cosmetic ingredients. Representative compounds include sec-butylbenzene, *m*-cymene, and *p*-cymene.

Finally, cluster 7 (Chi1n = 8.82, Chi0n = 13.46, NumValenceElectrons ≈ 115, SlogP–VSA5 = 102.68, Kappa1 = 18.62) is composed of long-chain organic compounds with some degree of branching, suggesting potential applications as synthesis intermediates, components of natural resins, or bioactive precursors. Representative molecules include trihexylamine, 1,1-difluoroheptadecane, and heptadecanoic acid.

This general analysis highlights that the combined use of topological indices, molecular descriptors, and unsupervised learning techniques enables effective segmentation of chemical space and allows the generation of well-founded hypotheses regarding the function, reactivity, and applicability of organic compounds.

## Conclusions

This research work proposes a comprehensive strategy for the prediction of the standard enthalpy of combustion of organic compounds, employing topological indices derived from molecular graphs in combination with interpretable machine learning models. The study demonstrates that the Estrada, Gutman, and Wiener indices are efficient predictors, achieving evaluation metric values comparable to those obtained using conventional molecular descriptors. This highlights that topological indices encapsulate electronic, geometric, and structural/topological information of molecules. Additionally, specific models were developed for certain chemical families, achieving *R* ^2^ values close to 0.99, indicating that differentiated modeling approaches can yield more accurate predictions based on structural features. Furthermore, the use of nonparametric models combined with explainable artificial intelligence tools enabled the identification of the relative importance of each descriptor, supporting their applicability in molecular design tasks.

Clustering analysis within the chemical space defined by the Estrada, Gutman, and Wiener indices revealed latent structures and provided a new way to organize compounds based on their topological characteristics. However, it is important to note that this study has certain limitations—for instance, the generalization to underrepresented compounds or functional groups remains to be further explored, as does the influence of three-dimensional molecular conformation. As a continuation of this research, we propose incorporating the analysis of line graphs *L*(*G*), *L* ^2^(*G*), and *L* ^3^(*G*) of each molecule to include additional spectral and conformational descriptors, and extending the strategy to heteroatomic and organometallic compounds. This would contribute to the development of interactive platforms applicable in various contexts. Finally, it is worth emphasizing that the strategy proposed in this work is not limited to the prediction of standard combustion enthalpy, but can be adapted to construct QSPR models for various physicochemical, toxicity, or bioavailability properties.

## Supplementary Material





## Data Availability

The Python scripts used in this research, as well as the data sets, can be found in the following GitHub repository: https://github.com/jarzolads/PredictionEnthalpy

## Notes

SI_ACS_JCIM_22_05_24 (ZIP)
Includes Table S1, which provides a detailed description and classification of the molecular descriptors obtained using RDKit. Figure S1 illustrates the linear relationship observed between the standard enthalpy of combustion and the Estrada index across different classes of organic compounds. Figures S2 and S3 depict the nonlinear relationships between the standard enthalpy of combustion and the Wiener and Gutman indices, respectively. Table S2 presents a comparison of evaluation metrics for the validation of various regression models using topological indices as predictors of the standard enthalpy of combustion. Similarly, Table S3 reports the corresponding results obtained using RDKit-derived molecular descriptors. Table S4 presents the descriptive statistics for the topological indices and the standard enthalpy of combustion within each cluster, while Table S5 provides the descriptive statistics for each cluster based on the five molecular descriptors most strongly correlated with the topological indices (PDF)
