# Supplementary figures and images for: Prediction of Standard Combustion Enthalpy of Organic Compounds Combining Machine Learning and Chemical Graph Theory: A Strategy
Source: ACS Omega. 2025 Sep 8;10(36):41828–48. doi: 10.1021/acsomega.5c05927 (PMC12444530; doi:10.1021/acsomega.5c05927)

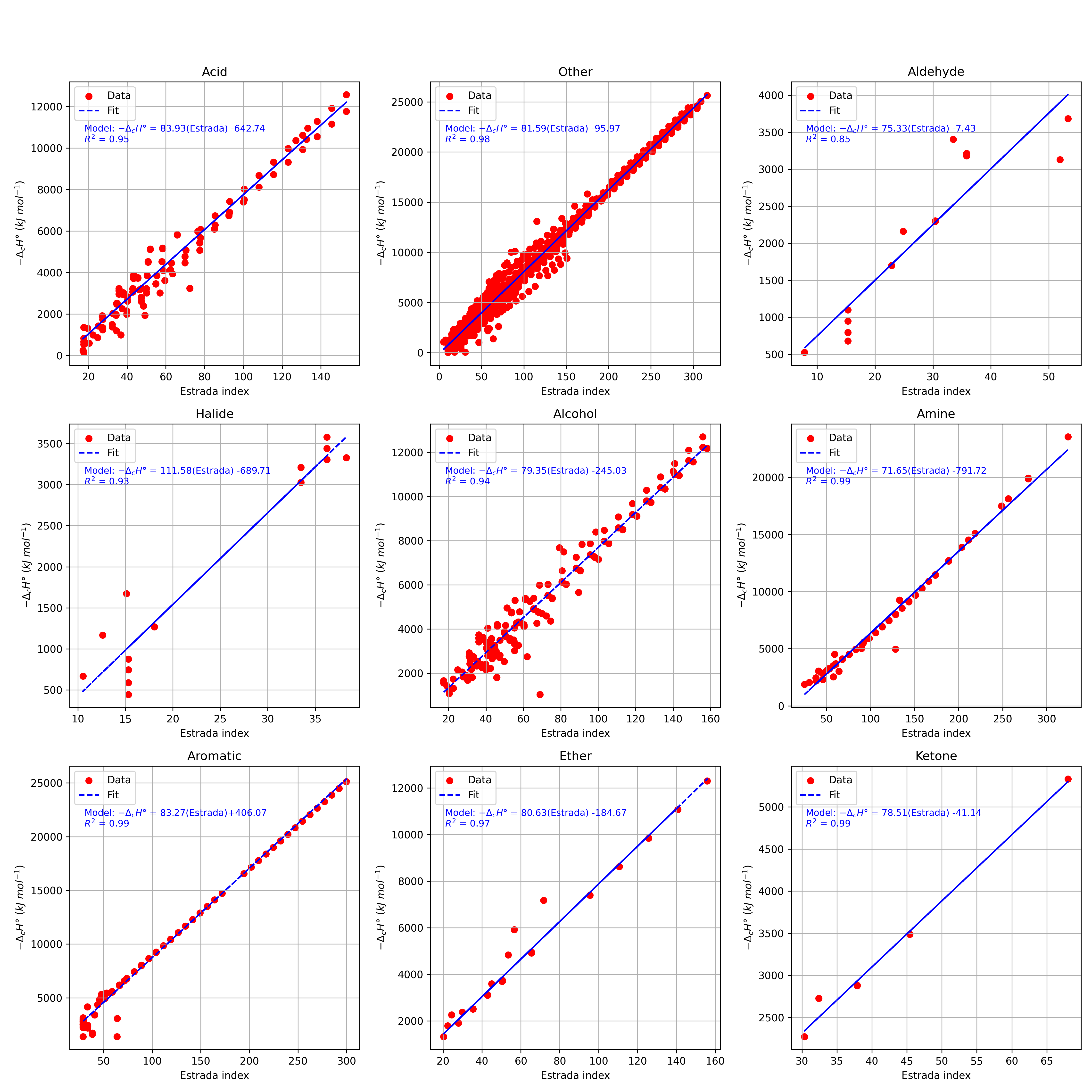

Supplement: Supplementary file 1 [file ao5c05927_si_001.zip › SI_ACS_JCIM_22_05_24/FigureS1-SI.png]
