# Supplementary material for: Prediction of Standard Combustion Enthalpy of Organic Compounds Combining Machine Learning and Chemical Graph Theory: A Strategy
Source: ACS Omega. 2025 Sep 8;10(36):41828–48. doi: 10.1021/acsomega.5c05927 (PMC12444530; doi:10.1021/acsomega.5c05927)
Supplement: Supplementary file 2 [file ao5c05927_si_002.pdf]

Prediction of standard combustion enthalpy of organic compounds combining machine learning and chemical graph theory: a strategy

Fernanda Saviñon-Flores, Jesús A. Arzola Flores,* Miguel A. García-Castro, Fausto Díaz-Sánchez, Esmeralda Vidal Robles, and Fidel Aaron Maruri Valderrabano

Facultad de Ingeniería Química de la Benemérita Universidad Autónoma de Puebla, 18 Sur y Av. San Claudio, C.P. 72560, Puebla Pue, México

E-mail: jesus.arzolaflores@correo.buap.mx

Phone: +052 2212038067

Supporting Information Available

LIST OF CONTENTS

Table S1: RDKit descriptors description and classification.

Table S2: Comparison of evaluation metrics for the validation process across different regression models, using topological indices as predictors of the standard enthalpy of combustion.

Table S3: Comparison of evaluation metrics for the validation process across different regression models, using the molecular descriptors obtained from RDKit as predictors of the standard enthalpy of combustion.

Table S4: Descriptive statistics and confidence intervals (95% and 99%) for each variable across clusters.

Table S5: Descriptive statistics and confidence intervals (95% and 99%) for the top five correlations from Figure 11 for each cluster.

Figure S1: Standard combustion enthalpy as a function of the Estrada index for different families of organic compounds. In all cases, a linear relationship is observed.

Figure S2: Standard combustion enthalpy as a function of the Wiener index for different families of organic compounds. In all cases, a linear relationship is observed.

Figure S3: Standard combustion enthalpy as a function of the Gutman index for different families of organic compounds. In all cases, a linear relationship is observed.

Table S1: RDKid descriptors description and classification[1–21]

Descriptor Name	Descriptor Classification	Descriptor Type	Description
MaxAbsEStateIndex	1D	Continuous	Returns a tuple of State indices for the molecule
MaxEStateIndex	1D	Continuous	Returns a tuple of State indices for the molecule
MinAbsEStateIndex	1D	Continuous	Returns a tuple of State indices for the molecule
MinEStateIndex	1D	Continuous	Returns a tuple of State indices for the molecule
qed	2D	Continuous	Combines eight physicochemical properties (molecular weight, LogP, H-bond donors, H-bond acceptors, charge, aromaticity, stereochemistry, and solubility), generating a score between 0 and 1
SPS	2D	Continuous	Score system to express the spatial complexity of a compound in a uniform manner and on a highly granular scale for ranking and comparison between molecules
MolWt	2D	Continuous	The average molecular weight of the molecule

Descriptor Name	Descriptor Classification	Descriptor Type	Description
HeavyAtomMolWt	2D	Continuous	The average molecular weight of the molecule ignoring hydrogens
ExactMolWt	2D	Continuous	The exact molecular weight of the molecule
NumValenceElectrons	1D	Continuous	The number of valence electrons the molecule has
NumRadicalElectrons	2D	Continuous	The number of radical electrons the molecule has (says nothing about spin state)
MaxPartialCharge	1D	Continuous	Returns a maximum partial charge as a non-integer charge value when measured in elementary charge units
MinPartialCharge	1D	Continuous	Returns a minimal partial charge as a non-integer charge value when measured in elementary charge units
MaxAbsPartialCharge	1D	Continuous	Returns a maximum absolute partial charge as a non-integer charge value when measured in elementary charge units

Descriptor Name	Descriptor Classification	Descriptor Type	Description
MinAbsPartialCharge	1D	Continuous	Returns a minimal absolute partial charge as a non-integer charge value when measured in elementary charge units
FpDensityMorgan1	2D	Fingerprint	Returns Morgan fingerprint density
FpDensityMorgan2	2D	Fingerprint	Returns Morgan fingerprint density
FpDensityMorgan3	2D	Fingerprint	Returns Morgan fingerprint density
BCUT2D$_MWHI$	2D	Fingerprint	Returns a 2D BCUT (eigenvalue high) given the molecule and the specified atom props
BCUT2D$_MWLOW$	2D	Fingerprint	Returns a 2D BCUT (eigenvalue low) given the molecule and the specified atom props
BCUT2D$_CHGHI$	2D	Fingerprint	Returns a 2D BCUT highest eigenvalue weighted by gasteiger charges
BCUT2D$_CHGLO$	2D	Fingerprint	Returns a 2D BCUT lowest eigenvalue weighted by gasteiger charges

**Descriptor Name**	**Descriptor Classification**	**Descriptor Type**	**Description**
BCUT2D$_LOGPHI$	2D	Fingerprint	Returns a 2D BCUT highest eigenvalue weighted by crippen logP
BCUT2D$_LOGPLOW$	2D	Fingerprint	Returns a 2D BCUT lowest eigenvalue weighted by crippen logP
BCUT2D$_MRHI$	2D	Fingerprint	Returns a 2D BCUT highest eigenvalue weighted by crippen MRR
BCUT2D$_MRLOW$	2D	Fingerprint	Returns a 2D BCUT lowest eigenvalue weighted by crippen MRR
Avglpc	2D	Continuous	Returns the average information content of the coefficients of the characteristic polynomial of the adjacency matrix of a hydrogen-suppressed graph of a molecule
BalabanJ	2D	Continuous	Balaban's J value for a molecule
BertzCT	2D	Continuous	A topological index meant to quantify complexity of molecules

**Descriptor Name**	**Descriptor Classification**	**Descriptor Type**	**Description**
Chi0	2D	Continuous	Returns molecular connectivity chi index that characterize the structural attributes of molecule of kappa indexes of molecular shape
Chi0n	2D	Continuous	Similar to Hall Kier Chi0v, returns the ChiXn value for a molecule for X=0-4
Chi0v	2D	Continuous	Connectivity descriptor that returns the ChiXv value for a molecule for X=0-4
Chi1	2D	Continuous	Returns molecular connectivity chi index that characterize the structural attributes of molecule of kappa index of molecular shape equal to 1
Chi0	2D	Continuous	Returns molecular connectivity chi index that characterizes the structural attributes of the molecule
Chi0n	2D	Continuous	Similar to Hall Kier Chi0v, returns the ChiXn value for a molecule for X=0-4

**Descriptor Name**	**Descriptor Classification**	**Descriptor Type**	**Description**
Chi0v	2D	Continuous	Connectivity descriptor that returns the ChiXv value for a molecule for X=0-4
Chi1n	2D	Continuous	Similar to Hall Kier Chi0v, returns the ChiXn value for a molecule for X=0-4
Chi1v	2D	Continuous	Connectivity descriptor that returns the ChiXv value for a molecule for X=0-4
Chi2n	2D	Continuous	Similar to Hall Kier Chi2v, but uses nVal instead of valence. This makes a big difference after we get out of the first row
Chi2v	2D	Continuous	Connectivity descriptor that returns the ChiXv value for a molecule for X=0-4
Chi3n	2D	Continuous	Similar to Hall Kier Chi3v, but uses nVal instead of valence. This makes a big difference after we get out of the first row

Descriptor Name	Descriptor Classification	Descriptor Type	Description
Chi3v	2D	Continuous	Connectivity descriptor that returns the ChiXv value for a molecule for X=0-4
Chi4n	2D	Continuous	Similar to Hall Kier Chi4v, but uses nVal instead of valence. This makes a big difference after we get out of the first row
Chi4v	2D	Continuous	Connectivity descriptor that returns the ChiXv value for a molecule for X=0-4
HallKierAlpha	2D	Continuous	The Hall-Kier alpha returns the presence of C(sp3) or non-C(sp3) atoms, modifying each kappa index with the influence of the covalent radius on the shape modeled
Ipc	2D	Continuous	The information content of the coefficients of the characteristic polynomial of the adjacency matrix of a hydrogen-suppressed graph of a molecule

**Descriptor Name**	**Descriptor Classification**	**Descriptor Type**	**Description**
Kappa1	2D	Continuous	Kappa indices are calculated relative to the least branched (linear) and most branched (star) compounds with the same number of atoms as the molecule being investigated
Kappa2	2D	Continuous	Hall-Kier Kappa2 value
Kappa3	2D	Continuous	Hall-Kier Kappa3 value
LabuteASA	3D	Continuous	Labute's Approximate Surface Area (ASA from MOE)
PEOE_VSA1	3D	Fingerprint	MOE (Molecular Operating Environment) Charge VSA Descriptor 1 (-inf < x < -0.30)
PEOE_VSA10	3D	Fingerprint	MOE Charge VSA Descriptor
PEOE_VSA11	3D	Fingerprint	MOE Charge VSA Descriptor 11 (0.15 <= x < 0.20)
PEOE_VSA12	3D	Fingerprint	MOE Charge VSA Descriptor 12 (0.20 <= x < 0.25)
PEOE_VSA13	3D	Fingerprint	MOE Charge VSA Descriptor 13 (0.25 <= x < 0.30)
PEOE_VSA14	3D	Fingerprint	MOE Charge VSA Descriptor 14 (0.30 <= x < inf)

Descriptor Name	Descriptor Classification	Descriptor Type	Description
PEOE_VSA2	3D	Fingerprint	MOE Charge VSA Descriptor 2 (-0.30 <= x < -0.25)
PEOE_VSA3	3D	Fingerprint	MOE Charge VSA Descriptor 3 (-0.25 <= x < -0.20)
PEOE_VSA4	3D	Fingerprint	MOE Charge VSA Descriptor 4 (-0.20 <= x < -0.15)
PEOE_VSA5	3D	Fingerprint	MOE Charge VSA Descriptor 5 (-0.15 <= x < -0.10)
PEOE_VSA6	3D	Fingerprint	MOE Charge VSA Descriptor 6 (-0.10 <= x < -0.05)
PEOE_VSA7	3D	Fingerprint	MOE Charge VSA Descriptor 7 (-0.05 <= x < 0.00)
PEOE_VSA8	3D	Fingerprint	MOE Charge VSA Descriptor 8 (0.00 <= x < 0.05)
PEOE_VSA9	3D	Fingerprint	MOE Charge VSA Descriptor 9 (0.05 <= x < 0.10)
SMR_VSA1	3D	Fingerprint	MOE MR VSA Descriptor 1 (-inf < x < 1.29)
SMR_VSA10	3D	Fingerprint	MOE MR VSA Descriptor 10
SMR_VSA2	3D	Fingerprint	MOE MR VSA Descriptor 2 (1.29 <= x < 1.82)
SMR_VSA3	3D	Fingerprint	MOE MR VSA Descriptor 3 (1.82 <= x < 2.24)

**Descriptor Name**	**Descriptor Classification**	**Descriptor Type**	**Description**
SMR_VSA4	3D	Fingerprint	MOE MR VSA Descriptor 4 (2.24 <= x < 2.45)
SMR_VSA5	3D	Fingerprint	MOE MR VSA Descriptor 5 (2.45 <= x < 2.75)
SMR_VSA6	3D	Fingerprint	MOE MR VSA Descriptor 6 (2.75 <= x < 3.05)
SMR_VSA7	3D	Fingerprint	MOE MR VSA Descriptor 7 (3.05 <= x < 3.63)
SMR_VSA8	3D	Fingerprint	MOE MR VSA Descriptor 8 (3.63 <= x < 3.80)
SMR_VSA9	3D	Fingerprint	MOE MR VSA Descriptor 9 (3.80 <= x < 4.00)
SlogP_VSA1	3D	Fingerprint	MOE logP VSA Descriptor 1 (-inf < x < -0.40)
SlogP_VSA10	3D	Fingerprint	MOE logP VSA Descriptor 10 (0.40 <= x < 0.50)
SlogP_VSA11	3D	Fingerprint	MOE logP VSA Descriptor 11 (0.50 <= x < 0.60)
SlogP_VSA12	3D	Fingerprint	MOE logP VSA Descriptor 12 (0.60 <= x < inf)
SlogP_VSA2	3D	Fingerprint	MOE logP VSA Descriptor 2 (-0.40 <= x < -0.20)
SlogP_VSA3	3D	Fingerprint	MOE logP VSA Descriptor 3 (-0.20 <= x < 0.00)

Descriptor Name	Descriptor Classification	Descriptor Type	Description
SlogP_VSA4	3D	Fingerprint	MOE logP VSA Descriptor 4 (0.00 <= x < 0.10)
SlogP_VSA5	3D	Fingerprint	MOE logP VSA Descriptor 5 (0.10 <= x < 0.15)
SlogP_VSA6	3D	Fingerprint	MOE logP VSA Descriptor 6 (0.15 <= x < 0.20)
SlogP_VSA7	3D	Fingerprint	MOE logP VSA Descriptor 7 (0.20 <= x < 0.25)
SlogP_VSA8	3D	Fingerprint	MOE logP VSA Descriptor 8 (0.25 <= x < 0.30)
SlogP_VSA9	3D	Fingerprint	MOE logP VSA Descriptor 9 (0.30 <= x < 0.40)
TPSA	2D	Continuous	The polar surface area (PSA) or topological polar surface area (TPSA) of a molecule is defined as the surface sum over all polar atoms or molecules, primarily oxygen and nitrogen, also including their attached hydrogen atoms
EState_VSA1	1D	Fingerprint	EState VSA Descriptor 1 (-inf < x < -0.39)
EState_VSA10	1D	Fingerprint	EState VSA Descriptor 10 (9.17 <= x < 15.00)

Descriptor Name	Descriptor Classification	Descriptor Type	Description
EState_VSA11	1D	Fingerprint	EState VSA Descriptor 11 (15.00 <= x < inf)
EState_VSA2	1D	Fingerprint	EState VSA Descriptor 2 (-0.39 <= x < 0.29)
EState_VSA3	1D	Fingerprint	EState VSA Descriptor 3 (0.29 <= x < 0.72)
EState_VSA4	1D	Fingerprint	EState VSA Descriptor 4 (0.72 <= x < 1.17)
EState_VSA5	1D	Fingerprint	EState VSA Descriptor 5 (1.17 <= x < 1.54)
EState_VSA6	1D	Fingerprint	EState VSA Descriptor 6 (1.54 <= x < 1.81)
EState_VSA7	1D	Fingerprint	EState VSA Descriptor 7 (1.81 <= x < 2.05)
EState_VSA8	1D	Fingerprint	EState VSA Descriptor 8 (2.05 <= x < 4.69)
EState_VSA9	1D	Fingerprint	EState VSA Descriptor 9 (4.69 <= x < 9.17)
VSA_EState1	1D	Fingerprint	VSA EState Descriptor 1 (-inf < x < 4.78)
VSA_EState10	1D	Fingerprint	VSA EState Descriptor 10 (11.00 <= x < inf)
VSA_EState2	1D	Fingerprint	VSA EState Descriptor 2 (4.78 <= x < 5.00)

**Descriptor Name**	**Descriptor Classification**	**Descriptor Type**	**Description**
VSA_EState3	1D	Fingerprint	VSA EState Descriptor 3 (5.00 <= x < 5.41)
VSA_EState4	1D	Fingerprint	VSA EState Descriptor 4 (5.41 <= x < 5.74)
VSA_EState5	1D	Fingerprint	VSA EState Descriptor 5 (5.74 <= x < 6.00)
VSA_EState6	1D	Fingerprint	VSA EState Descriptor 6 (6.00 <= x < 6.07)
VSA_EState7	1D	Fingerprint	VSA EState Descriptor 7 (6.07 <= x < 6.45)
VSA_EState8	1D	Fingerprint	VSA EState Descriptor 8 (6.45 <= x < 7.00)
VSA_EState9	1D	Fingerprint	VSA EState Descriptor 9 (7.00 <= x < 11.00)
FractionCSP3	3D	Continuous	The fraction of C atoms that are SP3 hybridized.
HeavyAtomCount	2D	Continuous	Number of heavy atoms of a molecule
NHOHCount	2D	Continuous	Number of NHs and OHs
NOCount	2D	Continuous	Number of Nitrogen and Oxygen atoms

Descriptor Name	Descriptor Classification	Descriptor Type	Description
NumAliphaticCarbocycles	2D	Continuous	Returns the number of aliphatic (containing at least one non-aromatic bond) carbocycles for a molecule
NumAliphaticHeterocycles	2D	Continuous	Returns the number of aliphatic (containing at least one non-aromatic bond) heterocycles for a molecule
NumAliphaticRings	2D	Continuous	Returns the number of aliphatic (containing at least one non-aromatic bond) rings for a molecule
NumAromaticCarbocycles	2D	Continuous	The number of aromatic carbocycles for a molecule
NumAromaticHeterocycles	2D	Continuous	The number of aromatic heterocycles for a molecule
NumAromaticRings	2D	Continuous	The number of aromatic rings for a molecule
NumHAcceptors	2D	Continuous	Number of Hydrogen Bond Acceptors
NumHDonors	2D	Continuous	Number of Hydrogen Bond Donors
NumHeteroatoms	2D	Continuous	Number of Heteroatoms

Descriptor Name	Descriptor Classification	Descriptor Type	Description
NumRotatableBonds	2D	Continuous	Number of Rotatable Bonds
NumSaturatedCarbocycles	2D	Continuous	Returns the number of saturated carbocycles for a molecule
NumSaturatedHeterocycles	2D	Continuous	Returns the number of saturated heterocycles for a molecule
NumSaturatedRings	2D	Continuous	Number of Saturated Rings
RingCount	2D	Continuous	Number of All Rings
MolLogP	2D	Continuous	Returns solvent/water partition coefficient (Wildman-Crippen LogP value)
MolMR	3D	Continuous	Molar Refractivity(Wildman-Crippen MR value)
fr_Al_COO	2D	Continuous	Number of aliphatic carboxylic acids
fr_Al_OH	2D	Continuous	Number of aliphatic hydroxyl groups
fr_Al_OH_noTert	2D	Continuous	Number of aliphatic hydroxyl groups excluding tert-OH
fr_ArN	2D	Continuous	Number of N functional groups attached to aromatics

Descriptor Name	Descriptor Classification	Descriptor Type	Description
fr_Ar_COO	2D	Continuous	Number of Aromatic carboxylic acide
fr_Ar_N	2D	Continuous	Number of aromatic nitrogens
fr_Ar_NH	2D	Continuous	Number of aromatic amines
fr_Ar_OH	2D	Continuous	Number of aromatic hydroxyl groups
fr_COO	2D	Continuous	Number of carboxylic acids
fr_COO2	2D	Continuous	Number of carboxylic acids
fr_C_O	2D	Continuous	Number of carbonyl O
fr_C_O_noCOO	2D	Continuous	Number of carbonyl O, excluding COOH
fr_C_S	2D	Continuous	Number of thiocarbonyl
fr_HOCCN	2D	Continuous	Number of C(OH)CCN-Ctert-alkyl or C(OH)CCNcyclic
fr_Imine	1D	Continuous	Number of Imines
fr_NH0	2D	Continuous	Number of Tertiary amines
fr_NH1	2D	Continuous	Number of Secondary amines
fr_NH2	2D	Continuous	Number of Primary amines
fr_N_O	2D	Continuous	Number of hydroxylamine groups
fr_Ndealkylation1	2D	Continuous	Number of XCCNR groups

Descriptor Name	Descriptor Classification	Descriptor Type	Description
fr_Ndealkylation2	2D	Continuous	Number of tert-alicyclic amines (no heteroatoms, not quinine-like bridged N)
fr_Nhpyrrole	2D	Continuous	Number of H-pyrrole nitrogens
fr_SH	2D	Continuous	Number of thiol groups
fr_aldehyde	2D	Continuous	Number of aldehydes
fr_alkyl_carbamate	2D	Continuous	Number of alkyl carbamates (subject to hydrolysis)
fr_alkyl_halide	2D	Continuous	Number of alkyl halides
fr_allylic_oxid	2D	Continuous	Number of allylic oxidation sites excluding steroid dienone
fr_amide	2D	Continuous	Number of amides
fr_amidine	2D	Continuous	Number of amidine groups
fr_aniline	2D	Continuous	Number of anilines
fr_aryl_methyl	2D	Continuous	Number of aryl methyl sites for hydroxylation
fr_azide	2D	Continuous	Number of azide groups
fr_azo	2D	Continuous	Number of azo groups
fr_barbitur	2D	Continuous	Number of barbiturate groups
fr_benzene	2D	Continuous	Number of benzene rings

Descriptor Name	Descriptor Classification	Descriptor Type	Description
fr_benzodiazepine	2D	Continuous	Number of benzodiazepines with no additional fused rings
fr_bicyclic	2D	Continuous	Number of Bicyclic groups
fr_diazo	2D	Continuous	Number of diazo groups
fr_dihydropyridine	2D	Continuous	Number of dihydropyridines
fr_epoxide	2D	Continuous	Number of epoxide rings
fr_ester	2D	Continuous	Number of esters
fr_ether	2D	Continuous	Number of ether oxygens (including phenoxy)
fr_furan	2D	Continuous	Number of furan rings
fr_guanido	2D	Continuous	Number of guanidine groups
fr_halogen	2D	Continuous	Number of halogens
fr_hdrzine	2D	Continuous	Number of hydrazine groups
fr_hdrzone	2D	Continuous	Number of hydrazone groups
fr_imidazole	2D	Continuous	Number of imidazole rings
fr_imide	2D	Continuous	Number of imide groups
fr_isocyan	2D	Continuous	Number of isocyanates
fr_isothiocyan	2D	Continuous	Number of isothiocyanates
fr_ketone	2D	Continuous	Number of ketones

Descriptor Name	Descriptor Classification	Descriptor Type	Description
fr_ketone_Topliss	2D	Continuous	Number of ketones excluding diaryl, a,b-unsat. dienones, heteroatom on Calpha
fr_lactam	2D	Continuous	Number of beta lactams
fr_lactone	2D	Continuous	Number of cyclic esters (lactones)
fr_methoxy	2D	Continuous	Number of methoxy groups -OCH3
fr_morpholine	2D	Continuous	Number of morpholine rings
fr_nitrile	2D	Continuous	Number of nitriles
fr_nitro	2D	Continuous	Number of nitro groups
fr_nitro_arom	2D	Continuous	Number of nitro benzene ring substituents
fr_nitro_arom_nonortho	2D	Continuous	Number of non-ortho nitro benzene ring substituents
fr_nitroso	2D	Continuous	Number of nitroso groups, excluding NO2
fr_oxazole	2D	Continuous	Number of oxazole rings
fr_oxime	2D	Continuous	Number of oxime groups
fr_para_hydroxylation	2D	Continuous	Number of para-hydroxylation sites
fr_phenol	2D	Continuous	Number of phenols

Descriptor Name	Descriptor Classification	Descriptor Type	Description
fr_phenol_noOrthoHbond	2D	Continuous	Number of phenolic OH excluding ortho intramolecular Hbond substituents
fr_phos_acid	2D	Continuous	Number of phosphoric acid groups
fr_phos_ester	2D	Continuous	Number of phosphoric ester groups
fr_piperdine	2D	Continuous	Number of piperdine rings
fr_piperzine	2D	Continuous	Number of piperzine rings
fr_priamide	2D	Continuous	Number of primary amides
fr_prisulfonamd	2D	Continuous	Number of primary sulfonamides
fr_pyridine	2D	Continuous	Number of pyridine rings
fr_quatN	2D	Continuous	Number of quarternary nitrogens
fr_sulfide	2D	Continuous	Number of thioether
fr_sulfonamd	2D	Continuous	Number of sulfonamides
fr_sulfone	2D	Continuous	Number of sulfone groups
fr_term_acetylene	2D	Continuous	Number of terminal acetylenes
fr_tetrazole	2D	Continuous	Number of tetrazole rings
fr_thiazole	2D	Continuous	Number of thiazole rings
fr_thiocyan	2D	Continuous	Number of thiocyanates
fr_thiophene	2D	Continuous	Number of thiophene rings

Descriptor Name	Descriptor Classification	Descriptor Type	Description
fr_unbrch_alkane	2D	Continuous	Number of unbranched alkanes of at least 4 members (excludes halogenated alkanes)
fr_urea	2D	Continuous	Number of urea groups

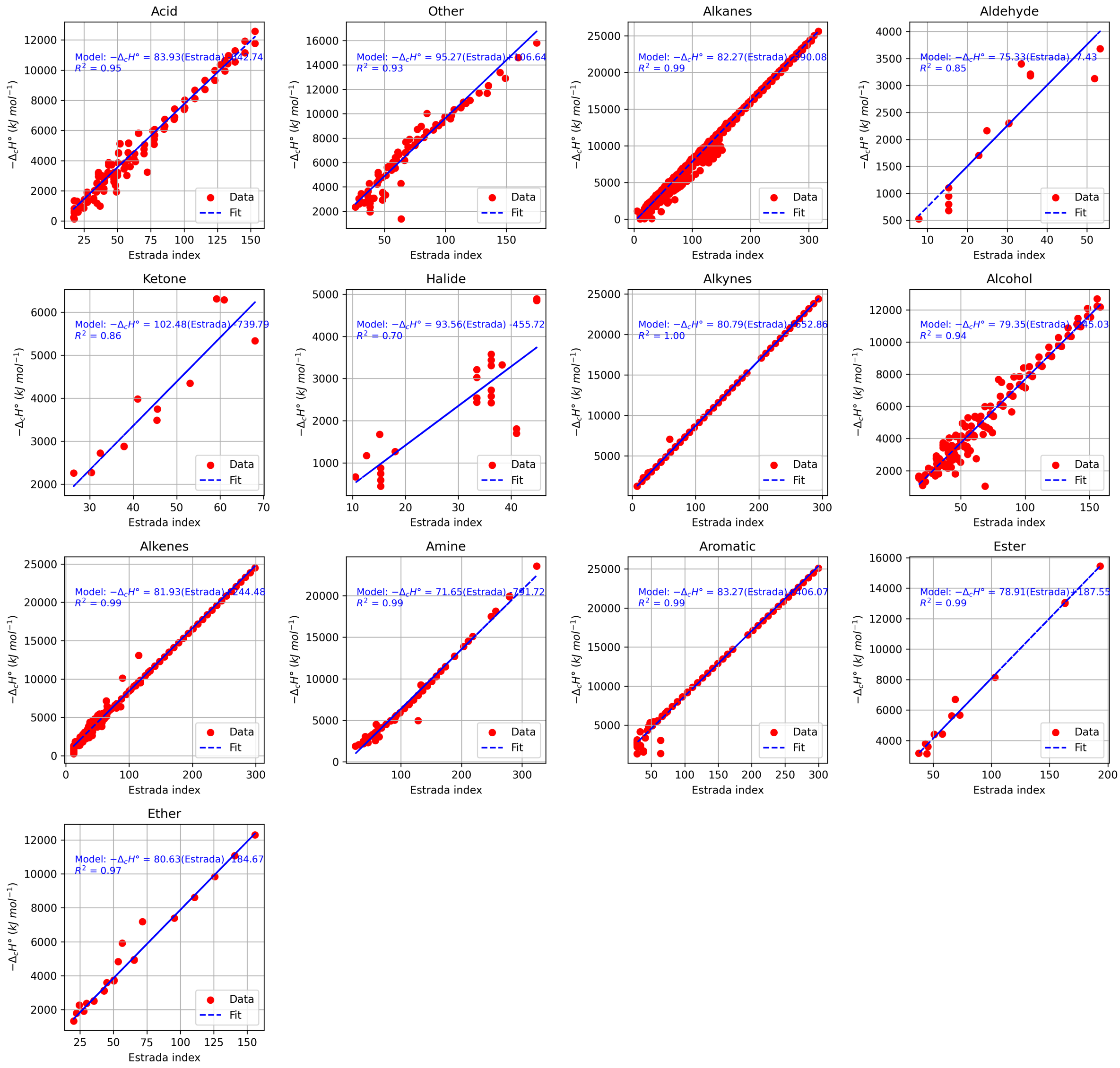

Figure S1: Standard combustion enthalpy as a function of the Estrada index for different families of organic compounds. In all cases, a linear relationship is observed.

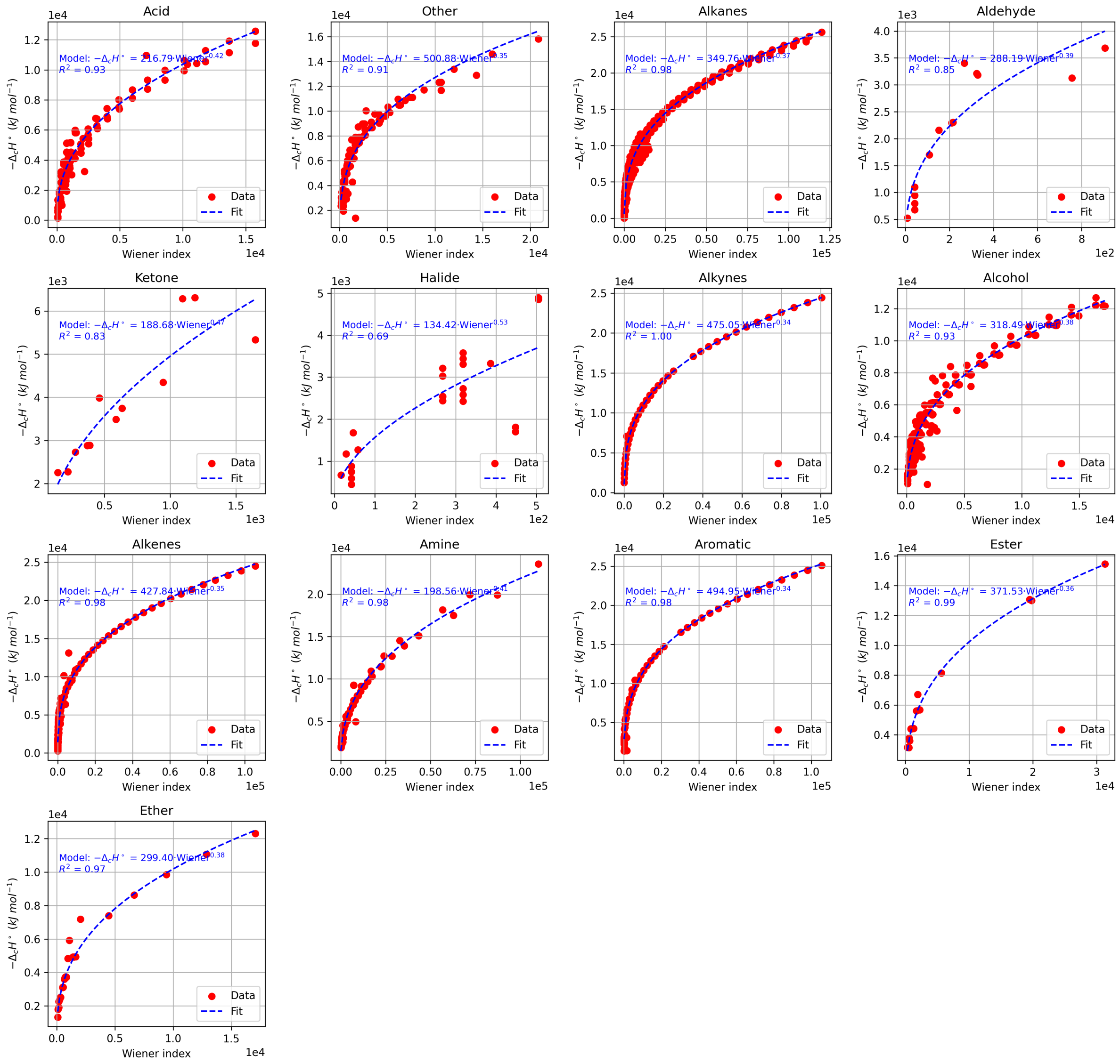

Figure S2: Standard combustion enthalpy as a function of the Wiener index for different families of organic compounds. In all cases, a nonlinear relationship is observed.

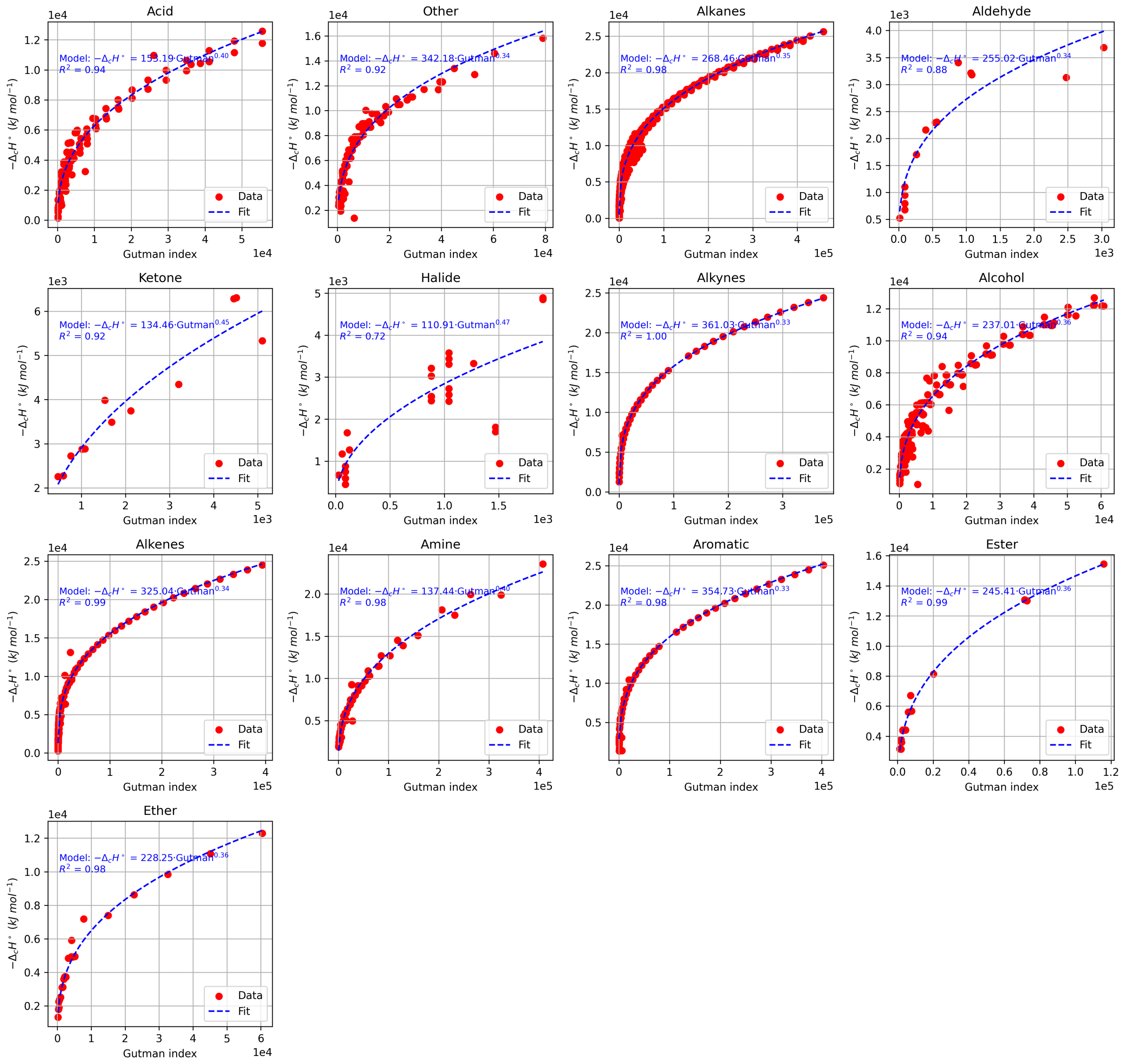

Figure S3: Standard combustion enthalpy as a function of the Gutman index for different families of organic compounds. In all cases, a nonlinear relationship is observed.

Table S2: Comparison of evaluation metrics for the validation process across different regression models, using topological indices as predictors of the standard enthalpy of combustion.

Model	MAE	MSE	RMSE	R^2	RMSLE	MAPE	TT (Sec)
Random Forest Regressor	266.5924	249271.4342	493.9055	0.9863	0.1856	0.1121	1.1430
Extra Trees Regressor	262.7963	251526.7031	497.4291	0.9861	0.1884	0.1125	0.6730
Light Gradient Boosting Machine	299.6606	252117.2368	498.6294	0.9860	0.1857	0.1222	0.9470
Gradient Boosting Regressor	329.9974	269110.8590	515.8759	0.9850	0.1902	0.1326	0.4380
Extreme Gradient Boosting	290.8954	272132.9328	516.8076	0.9850	0.1893	0.1136	0.2320
K Neighbors Regressor	343.9863	328074.6750	568.5800	0.9818	0.1991	0.1319	0.0510
Linear Regression	401.0880	335453.0094	577.2594	0.9811	0.2319	0.1424	0.6950
Bayesian Ridge	400.7349	335564.7469	577.3709	0.9811	0.2402	0.1424	0.0580
Huber Regressor	393.0971	341538.6454	582.5612	0.9807	0.2528	0.1437	0.0960
Ridge Regression	405.2619	352364.4375	591.7118	0.9802	0.2187	0.1489	0.0230
Lasso Regression	407.9876	357406.7562	595.9157	0.9799	0.2187	0.1496	0.0250
Lasso Least Angle Regression	408.1614	357575.3031	596.0590	0.9799	0.2189	0.1499	0.0450
Passive Aggressive Regressor	401.9362	363869.8441	601.4236	0.9795	0.2559	0.1490	0.0540
Least Angle Regression	423.4859	388413.4438	620.0244	0.9782	0.2375	0.1593	0.0430
Decision Tree Regressor	303.5847	397643.3750	624.0874	0.9781	0.2323	0.1177	0.0360
Orthogonal Matching Pursuit	453.5186	443678.3031	663.9203	0.9751	0.2226	0.1682	0.0370
AdaBoost Regressor	572.3171	568720.8316	752.0673	0.9678	0.2678	0.2257	0.2190
Elastic Net	755.7930	1009325.4750	1001.5750	0.9432	0.3102	0.2675	0.0230
Dummy Regressor	2960.7580	18068220.0000	4235.8408	-0.0058	0.7278	1.0210	0.0540

Table S3: Comparison of evaluation metrics for the validation process across different regression models, using the molecular descriptors obtained from RDKit as predictors of the standard enthalpy of combustion.

Model	MAE	MSE	RMSE	R^2	RMSLE	MAPE	TT (sec)
Ridge Regression	88.1622	35847.1068	187.5298	0.9979	0.1119	0.0349	0.0730
Bayesian Ridge	87.6409	36193.5871	188.1646	0.9979	0.1132	0.0340	0.4300
Lasso Regression	83.1790	38589.8146	193.4054	0.9977	0.1110	0.0286	0.1830
Lasso Least Angle Regression	90.3621	43650.7334	205.7263	0.9974	0.0995	0.0312	0.1070
Light Gradient Boosting Machine	114.4277	62977.0489	242.1699	0.9963	0.1180	0.0515	7.1920
Gradient Boosting Regressor	146.0968	64222.7262	248.2186	0.9962	0.1190	0.0580	4.2990
Orthogonal Matching Pursuit	146.1678	64678.5422	250.1600	0.9962	0.1403	0.0533	0.1280
Extra Trees Regressor	99.3120	72715.3038	262.5477	0.9957	0.1112	0.0543	5.4490
Elastic Net	165.4858	76394.7293	274.7316	0.9956	0.1142	0.0522	0.1550
Extreme Gradient Boosting	131.1455	80708.9734	280.5212	0.9953	0.1085	0.0550	1.8350
Random Forest Regressor	143.6763	108655.6922	325.4404	0.9936	0.1261	0.0661	15.1350
Passive Aggressive Regressor	93.5959	167861.5436	382.3424	0.9910	0.1308	0.0382	0.1250
Huber Regressor	81.4193	176091.4341	389.8160	0.9906	0.1028	0.0344	0.3680
Decision Tree Regressor	192.1558	243378.9897	482.0600	0.9861	0.1782	0.0736	0.2400
K Neighbors Regressor	434.1845	432628.4500	654.4188	0.9754	0.2118	0.1435	0.0980
AdaBoost Regressor	553.1689	474245.2309	688.2596	0.9728	0.2476	0.2074	1.7610
Dummy Regressor	2960.9552	18065197.2000	4234.3835	-0.0041	0.7268	1.0211	0.0780
Linear Regression	3012.6567	10438988259.4301	45448.9486	-630.4881	0.2680	2.2275	0.8660
Least Angle Regression	8.4e+23	inf	inf	-inf	42.0015	3.5e+23	0.1270

Table S4: Descriptive statistics and confidence intervals (95% and 99%) for each variable across clusters.

Cluster	Variable	Count	Mean	Std Dev	Min	25%	Median	75%	Max	95% CI Lower	95% CI Upper	99% CI Lower	99% CI Upper
1	Estrada	796	88.12	10.62	63.95	80.54	85.84	93.37	128.26	87.38	88.86	87.15	89.09
	Wiener	796	3220.09	1223.93	1271.00	2408.50	3000.50	3583.00	8337.00	3134.93	3305.24	3108.07	3332.10
	Gutman	796	10566.95	4242.69	5154.00	7678.00	9545.50	11801.50	28095.00	10271.76	10862.13	10178.67	10955.23
	$-\Delta_c H^o$	796	7157.38	831.66	4963.92	6386.06	7156.11	7532.46	10126.88	7099.52	7215.25	7081.27	7233.50
2	Estrada	64	242.10	13.82	218.44	228.92	242.55	252.03	278.85	238.65	245.55	237.51	246.69
	Wiener	64	57030.50	8899.91	42976.00	48736.00	56831.00	63856.00	73603.00	54807.37	59253.63	54075.57	59985.43
	Gutman	64	211019.98	33744.06	156679.00	178291.00	208991.00	235324.00	272257.00	202590.97	219449.00	199816.34	222223.62
	$-\Delta_c H^o$	64	19572.39	1170.67	17489.07	18483.22	19586.45	20413.71	22044.60	19279.97	19864.81	19183.71	19961.07
3	Estrada	81	191.11	16.49	164.24	176.20	191.27	206.33	218.63	187.46	194.76	186.28	195.95
	Wiener	81	29434.98	7056.65	18831.00	23116.00	29176.00	36208.00	43479.00	27874.62	30995.33	27366.05	31503.90
	Gutman	81	106959.80	26590.40	69636.00	82594.00	105076.00	131302.00	158691.00	101080.18	112839.42	99163.82	114755.79
	$-\Delta_c H^o$	81	15269.40	1567.99	11437.03	14103.86	15254.09	16553.21	18381.98	14922.68	15616.11	14809.68	15729.11
4	Estrada	1045	33.98	9.43	5.36	27.61	35.14	40.65	68.90	33.40	34.55	33.22	34.73
	Wiener	1045	322.72	198.37	4.00	174.00	317.00	436.00	1776.00	310.67	334.76	306.88	338.55
	Gutman	1045	933.89	634.90	6.00	475.00	898.00	1216.00	6487.00	895.35	972.43	883.20	984.57
	$-\Delta_c H^o$	1045	2424.81	842.57	31.60	1846.16	2530.82	3037.35	4160.82	2373.66	2475.95	2357.55	2492.07
5	Estrada	55	286.16	13.84	266.57	274.11	284.74	296.70	324.03	282.42	289.90	281.18	291.15
	Wiener	55	92464.47	12121.74	75496.00	81811.00	90903.00	102844.00	120057.00	89187.51	95741.44	88100.40	96828.55
	Gutman	55	345787.05	46555.57	279406.00	303373.00	342083.00	383410.00	458340.00	333201.32	358372.79	329026.09	362548.02
	$-\Delta_c H^o$	55	23133.23	1171.13	19885.63	22115.07	23231.10	23892.05	25635.33	22816.63	23449.83	22711.61	23554.86
6	Estrada	1139	58.82	8.53	37.62	52.69	58.01	65.54	83.10	58.33	59.32	58.17	59.48
	Wiener	1139	1132.54	439.75	312.00	786.00	1065.00	1390.00	2979.00	1106.97	1158.10	1098.92	1166.16
	Gutman	1139	3560.85	1422.03	1201.00	2412.00	3343.00	4345.50	9705.00	3478.18	3643.52	3452.13	3669.57
	$-\Delta_c H^o$	1139	4693.36	781.99	2630.62	4113.91	4766.46	5379.50	7077.06	4647.90	4738.82	4633.58	4753.15
7	Estrada	297	136.04	13.94	107.10	123.49	135.58	146.08	165.88	134.44	137.63	133.94	138.13
	Wiener	297	11367.59	3385.87	5060.00	8521.00	11096.00	13680.00	19809.00	10980.94	11754.24	10858.24	11876.94
	Gutman	297	39922.51	12214.13	18632.00	29233.00	38826.00	47934.00	72776.00	38527.71	41317.31	38085.08	41759.93
	$-\Delta_c H^o$	297	10746.79	1277.00	7676.60	9737.30	10611.41	11682.33	14604.75	10600.96	10892.61	10554.68	10938.89

Table S5: Descriptive statistics and confidence intervals (95% and 99%) for the top five correlations from Figure 9 for each cluster.

Cluster	Variable	Mean	Std Dev	Min	25%	Median	75%	Max	95% CI Lower	95% CI Upper	99% CI Lower	99% CI Upper
1	Chi0n	9.1828	0.9348	7.0711	8.3868	9.1104	9.7760	12.7258	9.1178	9.2477	9.0973	9.2683
	Chi1n	5.4535	0.7392	4.1547	4.9142	5.3284	5.8081	7.9850	5.4021	5.5048	5.3859	5.521
	NumValenceElectrons	74.4698	9.7612	60.0000	68.0000	74.0000	78.0000	116.0000	73.7917	75.1480	73.5772	75.3625
	SlogP-VSA5	57.2193	16.9494	0.0000	52.8753	59.7991	66.2199	96.8152	56.0419	58.3968	55.6694	58.7693
	Kappa1	11.7324	1.8848	7.8353	10.9600	11.6150	12.1467	19.1900	11.6014	11.8633	11.5600	11.9047
2	Chi0n	23.1692	1.3267	21.0919	22.0787	23.2944	24.1905	26.7817	22.8441	23.4942	22.7413	23.5971
	Chi1n	15.8883	0.9147	14.3493	15.0113	15.9516	16.4867	18.0700	15.6642	16.1124	15.5933	16.1832
	NumValenceElectrons	196.5000	11.0640	176.0000	188.0000	197.0000	206.0000	224.0000	193.7893	199.2107	192.9319	200.0681
	SlogP-VSA5	196.9460	12.2768	166.5869	186.7067	196.4122	205.9692	219.3138	193.9382	199.9538	192.9867	200.9052
	Kappa1	32.1816	2.0414	28.0333	30.6875	32.1393	33.7325	36.9600	31.6814	32.6817	31.5232	32.8399
3	Chi0n	18.4525	1.5268	16.1421	16.9343	18.3485	19.7627	21.3573	18.1200	18.7850	18.0148	18.8902
	Chi1n	12.4972	1.1039	10.8493	11.4744	12.4744	13.4744	14.4713	12.2568	12.7376	12.1807	12.8136
	NumValenceElectrons	156.9136	12.9771	134.0000	146.0000	156.0000	168.0000	182.0000	154.0875	159.7397	153.1935	160.6337
	SlogP-VSA5	152.2151	16.2426	113.8600	141.7610	154.6026	167.4443	180.7888	148.6779	155.7524	147.5589	156.8714
	Kappa1	25.5962	2.2417	21.0435	23.7300	25.4800	27.2900	30.5800	25.1080	26.0844	24.9535	26.2388
4	Chi0n	4.0498	1.0368	0.0000	3.3737	4.1463	4.6701	10.0163	3.9870	4.1127	3.9671	4.1326
	Chi1n	2.0749	0.6338	0.0000	1.6512	2.1124	2.4712	4.7598	2.0364	2.1133	2.0243	2.1255
	NumValenceElectrons	38.9129	10.2339	8.0000	32.0000	38.0000	44.0000	120.0000	38.2924	39.5334	38.0961	39.7297
	SlogP-VSA5	12.7244	9.2095	0.0000	6.4208	13.3446	20.2683	38.5249	12.1661	13.2828	11.9894	13.4595
	Kappa1	6.1165	1.7377	0.0000	4.9666	6.0300	7.1700	17.8000	6.0112	6.2219	5.9779	6.2552
5	Chi0n	27.2972	1.3022	25.3345	26.2169	27.0332	28.2480	31.0244	26.9530	27.6413	26.8441	27.7502
	Chi1n	18.8080	0.9208	17.4142	17.9744	18.8493	19.4744	21.0700	18.5647	19.0513	18.4877	19.1283
	NumValenceElectrons	231.7818	10.9725	212.0000	224.0000	230.0000	240.0000	260.0000	228.8819	234.6817	227.9646	235.5990
	SlogP-VSA5	234.6921	12.8576	205.1118	225.2317	231.6525	244.4941	263.7566	231.2941	238.0902	230.2191	239.1651
	Kappa1	38.1243	1.9862	34.0278	36.7350	38.0250	39.5200	42.9600	37.5994	38.6492	37.4333	38.8152
6	Chi0n	6.4037	0.9008	3.9831	5.6171	6.4113	7.1213	10.1815	6.3514	6.4560	6.3349	6.4726
	Chi1n	3.6161	0.5747	2.4958	3.1088	3.5863	4.0897	5.4495	3.5828	3.6495	3.5722	3.6601
	NumValenceElectrons	53.5312	8.3757	34.0000	48.0000	54.0000	60.0000	96.0000	53.0447	54.0176	52.8909	54.1715
	SlogP-VSA5	31.4221	11.9756	0.0000	25.6833	32.6070	40.0337	58.7932	30.7266	32.1176	30.5066	32.3376
	Kappa1	7.9871	1.5542	3.0612	7.0000	7.9200	8.9600	14.1900	7.8968	8.0774	7.8683	8.1059
7	Chi0n	13.4632	1.3277	11.1924	12.3077	13.4768	14.3763	18.1682	13.3122	13.6142	13.2644	13.6620
	Chi1n	8.8271	0.9085	7.1582	8.0236	8.8493	9.5000	11.1354	8.7237	8.9304	8.6911	8.9631
	NumValenceElectrons	115.2189	11.9326	92.0000	104.0000	116.0000	124.0000	158.0000	113.8617	116.5760	113.4325	117.0053
	SlogP-VSA5	102.6823	16.2396	16.6904	91.4002	103.2361	116.0777	135.8431	100.8354	104.5293	100.2512	105.1135
	Kappa1	18.6219	2.2303	13.8035	16.9600	18.7000	20.4000	24.1982	18.3682	18.8755	18.2880	18.9558

References

(1) Gasteiger, J., Ed. *Handbook of Chemoinformatics: From Data to Knowledge in 4 Volumes*; Wiley-VCH Verlag GmbH & Co. KgaA: Weinheim, 2003.

(2) Bonchev, D.; Trinajstić, N. Information theory, distance matrix, and molecular branching. *J. Chem. Phys.* **1977**, *67*, 4517–4533.

(3) Bickerton, G. R.; Paolini, G. V.; Besnard, J.; Muresan, S.; Hopkins, A. L. Quantifying the chemical beauty of drugs. *Nat. Chem.* **2012**, *4*, 90–98.

(4) Katritzky, A. R.; Lobanov, V. S.; Karelson, M. QSPR: The Correlation and Quantitative Prediction of Chemical and Physical Properties from Structure. *Chem. Soc. Rev.* **1995**, *24*, 279–287.

(5) Krzyzanowski, A.; Pahl, A.; Grigalunas, M.; Waldmann, H. Spacial ScoreA Comprehensive Topological Indicator for Small-Molecule Complexity. *J. Med. Chem.* **2023**,

(6) Bonchev, D.; Rouvray, D. *Chemical Graph Theory: Introduction and Fundamentals*; Abacus Press/Gordon & Breach Science Publisher: New York USA, 1991.

(7) Bertz, S. H. The first general index of molecular complexity. *J. Am. Chem. Soc.* **1981**, *103*, 3599–3601.

(8) Balaban, A. T. Highly discriminating distance-based topological index. *Chem. Phys. Lett.* **1982**, *89*, 399–404.

(9) Landrum, G. Rdkit documentation. 2013; `https://media.readthedocs.org/pdf/rdkit/latest/rdkit.pdf`.

(10) Mitchell, J. B. Machine learning methods in chemoinformatics. *Wiley Interdiscip. Rev. Comput. Mol. Sci.* **2014**, *4*, 468–481.

(11) Dutschmann, T. M.; Schlenker, V.; Baumann, K. Chemoinformatic regression methods and their applicability domain. *Mol. Inform.* **2024**, *43*, e202400018.

(12) Hall, L. H.; Mohney, B.; Kier, L. B. The Electrotopological State: An Atom Index for QSAR. *J.Chem.Inf.Comput.Sci.* **1991**, *31*, 76–81.

(13) Hall, L. H.; Kier, L. B. The molecular connectivity chi indexes and kappa shape indexes in structure-property modeling. *Rev. Comput. Chem.* **1991**, *2*, 367–422.

(14) Hall, L. H.; Kier, L. B. *The Hall-Kier alpha value for a molecule*; 1991; Vol. 2; pp 367–422.

(15) Hall, L. H.; Kier, L. B. Issues in representation of molecular structure: The development of molecular connectivity. *J. Mol. Graph. Model.* **2001**, *20*, 4–18.

(16) Hu, Q. N.; Liang, Y. Z.; Yin, H.; Peng, X. L.; Fang, K. T. Structural interpretation of the topological index. 2. The molecular connectivity index, the Kappa index, and the atom-type E-State index. *J. Chem. Inf. Comput. Sci.* **2004**, *44*, 1193–1201.

(17) Labute, P. In *"Chemoinformatics: Concepts, Methods, and Tools for Drug Discovery*; Bajorath, J., Ed.; Humana Press: Totowa, NJ, 2004; pp 261–278.

(18) Estrada, E. On the Topological Sub-Structural Molecular Design (TOSS-MODE) in QSPR/QSAR and Drug Design Research. *SAR. QSAR. Environ. Res.* **2000**, *11*, 55–73.

(19) Kier, L. B.; Hall, L. H. In *Topological Indices and Related Descriptors in QSAR and QSPR*; Devillers, J., Balaban, A. T., Eds.; Gordon and Breach Science Publishers: Amsterdam, 1999; pp 445–489.

(20) RDKit Developers RDKit: Descriptors Module. 2024; `https://www.rdkit.org/docs/source/rdkit.Chem.Descriptors.html`, Accessed: 2024-06-16.

(21) Gou, Y.; others Machine learning based models for high-throughput classification of human pregnane X receptor activators. *Env. sci., Adv.* **2023**, *2*, 304–312.
